# Hepatocyte-specific NR5A2 deficiency induces pyroptosis and exacerbates non-alcoholic steatohepatitis by downregulating ALDH1B1 expression

**DOI:** 10.1038/s41419-024-07151-1

**Published:** 2024-10-23

**Authors:** Rong Zhao, Zizhen Guo, Kaikai Lu, Qian Chen, Farooq Riaz, Yimeng Zhou, Luyun Yang, Xiaona Cheng, Litao Wu, Kexin Cheng, Lina Feng, Sitong Liu, Xiaodan Wu, Minghua Zheng, Chunyan Yin, Dongmin Li

**Affiliations:** 1 Department of Biochemistry and Molecular Biology, School of Basic Medical Sciences, Xi’an Jiaotong University Health Science Center, Xi’an, Shaanxi P.R. China; 2 Department of Plastic and Reconstructive Surgery, Shanghai Ninth People’s Hospital, Shanghai Jiao Tong University School of Medicine, Shanghai, China; 3 School of Medicine, Xizang Minzu University, Xianyang, Shaanxi P.R. China; 4 Center for Cancer Immunology, Faculty of Pharmaceutical Sciences, Shenzhen Institute of Advanced Technology, Chinese Academy of Sciences (CAS), Shenzhen, China; 5 Department of Planned Immunization, Xi’an Center for Disease Control and Prevention, No. 599 Xiying Road, Yanta District, Xi’an, Shaanxi China; 6 GMU-GIBH Joint School of Life Sciences, The Guangdong-Hong Kong-Macau Joint Laboratory for Cell Fate Regulation and Diseases, Guangzhou Medical University, Guangzhou, China; 7 MAFLD Research Center, Department of Hepatology, The First Affiliated Hospital of Wenzhou Medical University, Wenzhou, China; 8 Key Laboratory of Diagnosis and Treatment for the Development of Chronic Liver Disease in Zhejiang Province, Wenzhou, China; 9 Department of Pediatrics, The Second Affiliated Hospital of Xi’an Jiaotong University, Xi’an, Shanxi China; 10 Department of Biochemistry and Molecular Biology & Institute of Molecular and Translational Medicine, School of Basic Medical Sciences, Xi’an Jiaotong University Health Science Center, Xi’an, Shaanxi P.R. China; 11 Key Laboratory of Environment and Genes Related to Diseases, Ministry of Education, Xi’an, Shaanxi P.R. China

**Keywords:** Metabolomics, Autoimmune hepatitis

## Abstract

Nonalcoholic steatohepatitis (NASH) is a prevalent chronic disease, yet its exact mechanisms and effective treatments remain elusive. Nuclear receptor subfamily 5 group A member 2 (NR5A2), a transcription factor closely associated with cholesterol metabolism in the liver, has been hindered from comprehensive investigation due to the lethality of NR5A2 loss in cell lines and animal models. To elucidate the role of NR5A2 in NASH, we generated hepatocyte-specific knockout mice for Nr5a2 (Nr5a2^HKO^) and examined their liver morphology across different age groups under a regular diet. Furthermore, we established cell lines expressing haploid levels of NR5A2 and subsequently reintroduced various isoforms of NR5A2. In the liver of Nr5a2^HKO^ mice, inflammation and fibrosis spontaneously emerged from an early age, independent of lipid accumulation. Pyroptosis occurred in NR5A2-deficient cell lines, and different isoforms of NR5A2 reversed this form of cell death. Our findings unveiled that inhibition of NR5A2 triggers pyroptosis, a proinflammatory mode of cell death primarily mediated by the activation of the NF-κB pathway induced by reactive oxygen species (ROS). As a transcriptionally regulated molecule of NR5A2, aldehyde dehydrogenase 1 family member B1 (ALDH1B1) participates in pyroptosis through modulation of ROS level. In conclusion, the diverse isoforms of NR5A2 exert hepatoprotective effects against NASH by maintaining a finely tuned balance of ROS, which is contingent upon the activity of ALDH1B1.

## Highlights

- NR5A2 hepatocyte knockout mice aggravate NASH through NF-κB pathway and pyroptosis.
- NR5A2 transcriptionally upregulates ALDH1B1, inhibiting ROS.
- ALDH1B1 rescue experiments underscored its significance in NR5A2 mediated pyroptosis.
- Distinct NR5A2 isoforms exert consistent effect on ALDH1B1 expression and pyroptosis.

## Graphical abstract

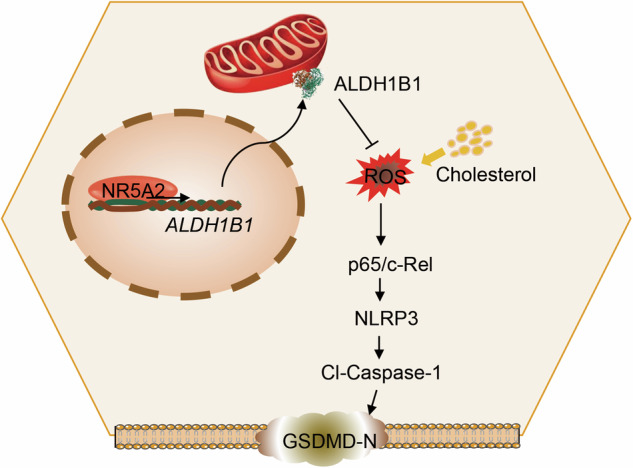

## Introduction

Nonalcoholic fatty liver disease (NAFLD), an inflammatory liver disease, exhibits a rising prevalence from 25.5% in or before 2005 to 37.8% in 2016 or later and has a 30% average global prevalence between 1990 and 2019 [1, 2]. Nonalcoholic steatohepatitis (NASH) is a reversible stage within NAFLD spectrum with an ambiguous pathogenic mechanism. Only one medication, Rezdiffra, has recently received approval from the Food and Drug Administration (FDA) for the treatment of NASH. This situation emphasizes the necessity for effective strategies to address NASH.

NR5A2 is a widely expressed transcription factor in vertebrate animals and plays a crucial role in embryonic survival [3, 4]. It has been reported that NR5A2 can participate in metabolism by modulating ingestion system and digestive system [5, 6]. A close relationship has been established between NR5A2 and cytochrome P450 family members, including CYP7A1, CYP8B1, CYP19, and CYP11a/b. These interactions play a crucial role in the synthesis of steroidal hormones and sterol metabolism within organisms [7–11]. Cholesteryl ester transfer protein (CETP), Scavenger receptor class B type I (SR-BI) and ATP binding cassette subfamily G member 5/8 (ABCG5/8) are cholesterol transporter molecules regulated by NR5A2 either independently or in cooperation with LXR/RXR and FXR [12–14]. Furthermore, Nr5a2 regulation of ELOVL fatty acid elongase 5 (Elovl5), Cyp8b1 and fatty acid desaturase 2 (Fads2) influences the arachidonoyl phospholipid pools in the livers of mice [15]. And impaired SUMOylation of NR5A2 has been found to promote NASH [16]. These researches supported that the NR5A2 played a crucial role in NASH.

NR5A2 has been proposed as a potential cancer marker due to its upregulation in tumors [17–19]. Suppression of NR5A2 leads to downregulation of cyclin E1 and subsequent apoptosis [20, 21]. These studies revealed that NR5A2 participated in lifecycle. In our study, we noticed that the cell death during incubation of NR5A2 knockout liver cell line may not be limited to apoptosis alone. Pyroptosis emerges as a promising avenue for further investigation linking NR5A2 and NASH.

Pyroptosis, also known as inflammatory necrosis, can accelerate the progression of NAFLD into an irreversible state [22, 23]. This mode of death is characterized by cellular swelling leading to membrane rupture and release of IL-1β [24]. The gasdermin family member, GSDMD, has been identified as a key player in the pyroptosis [25–27]. Additionally, the NF-κB pathway has been implicated in this inflammatory form of cell death [28, 29]. It has been suggested that NR5A2 indirectly regulates the NF-κB pathway through ERα, but there may be another regulatory pathway for the existence of mediate effect of NR5A2 on NF-κB pathway without Erα [30].

Subsequently, we identified ALDH1B1 as a target gene of NR5A2, which is widely recognized for its crucial role in alcohol metabolism as an acetaldehyde dehydrogenase. Notably, ALDH1B1 exhibits substrate activity towards lipid peroxide products such as malonaldehyde (MDA) and 4-hydroxynonenal (4-HNE) [31, 32], both of which are induced by reactive oxygen species (ROS) and can alter the fluidity and permeability of cell membrane [33]. ROS serves as a typical activator of the NF-κB pathway and pyroptosis [34, 35]. Lipid peroxide products have been found to positively regulate the NF-κB pathway [36]. Regarding pyroptosis, different perspectives exist. 4-HNE has been demonstrated to inhibit the activation of the NLRP3 inflammasome and macrophage pyroptosis [37]. However, it has also been observed that lipid peroxidation induces the formation of pores in the cell membrane through GSDMD-N bond with phospholipids [38]. ALDH2, which shares a sequence homology of 75% with Aldh1b1, has been investigated for its role in alleviating oxidative stress [31, 39]. Collectively, we hypothesize that deletion of NR5A2 may induce pyroptosis dependent on ALDH1B1.

## Results

### NR5A2 was downregulated in the liver of NASH patients and animal model

NR5A2, a crucial transcription factor involved in liver lipid metabolism, remains poorly understood in its association with NASH due to challenges posed by ineffective antibodies, the presence of different isoforms, and the cell survival issues associated with knockout models [6, 40]. In this study, we examined four human isoforms, three mouse isoforms, and three rat isoforms of NR5A2 through protein sequence alignment (Fig. S1A–S1C). Overexpression of various flag-tagged NR5A2 isoforms for human, mouse and rat was achieved in the LO2 cells. The NR5A2 antibody obtained from R&D SYSTEMS (PP-H2325-00) was chosen to identify the location of NR5A2 bands within the range of 50–70 kDa (Fig. S1D, S1E).

**Fig. 1:**
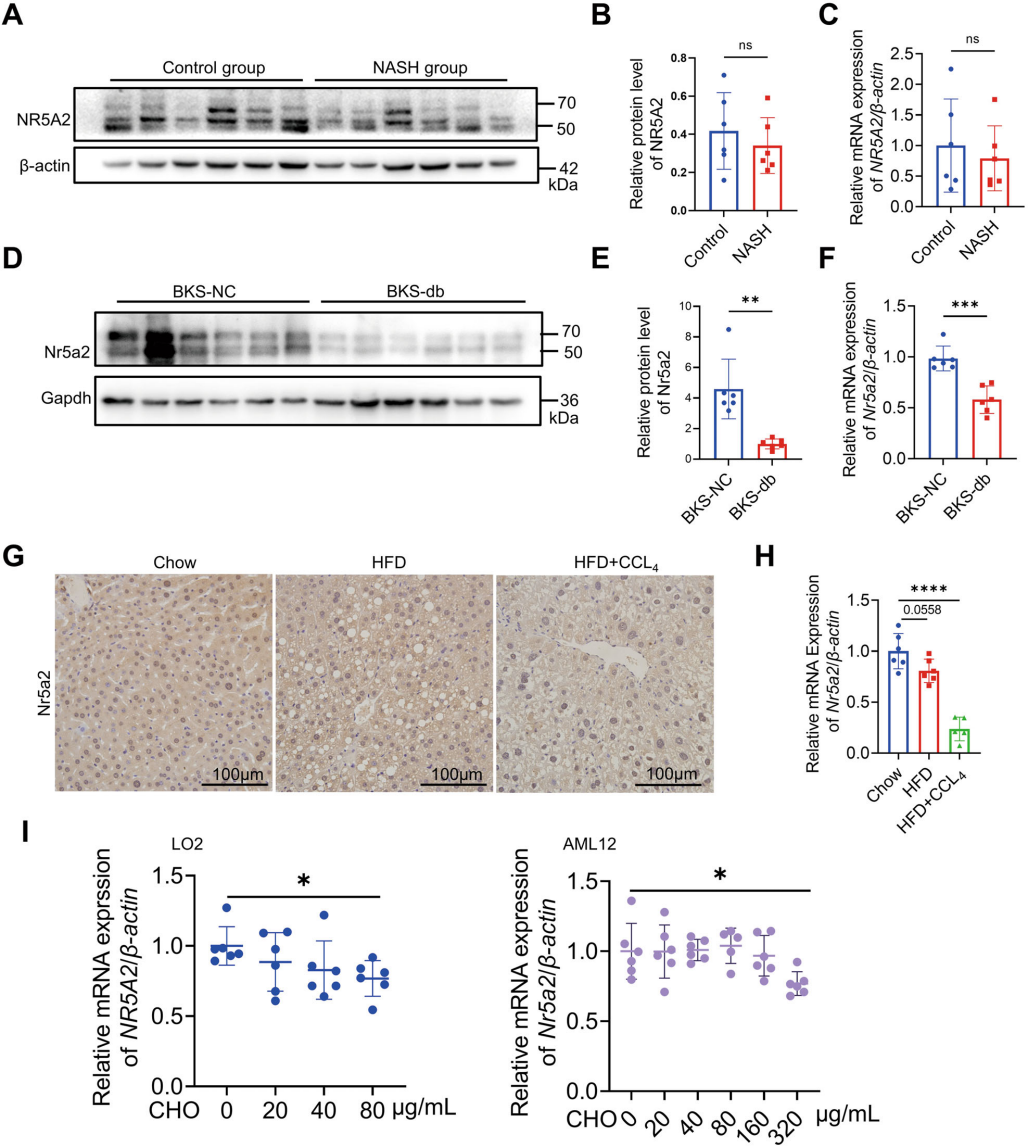
NR5A2 expression decreased in the liver of patients with NASH and mouse models of NASH. **A**–**C** Western blotting (**A**), statistical analysis (**B**), and RT-qPCR assay (**C**) were performed to evaluate the expression of NR5A2 in the livers of NASH patients and control group. *N* = 6/group, ns not significant. Data are presented as means ± SD. **D**–**F** Western blotting (**D**), statistical analysis (**E**), and RT-qPCR assay (**F**) were conducted to assess liver Nr5a2 expression in BKS-db mice and BKS-NC mice. *N* = 6/group, **P < 0.01, ***P < 0.001. Data are presented as means ± SD. **G**, **H** Immunohistochemical staining (**G**) and RT-qPCR assay (**H**) were employed to detect Nr5a2 expression in the HFD and HFD + CCL4 induced NASH mouse model. *N* = 5–6/group, ****P < 0.0001. Data are presented as means ± SD. **I** RT-qPCR was used to measure NR5A2 mRNA levels in LO2 and AML12 cell lines stimulated with different concentrations of cholesterol for 24 h. *N* = 6/group, *P < 0.05. Data are presented as means ± SD.

In patients with NASH, there was a declining trend observed in the expression of NR5A2, although no statistically significant differences were detected at either the transcriptional or protein level (Fig. 1A–C). Furthermore, Nr5a2 expression exhibited a significant decrease in the livers of BKS-db mice, which serve as a model of type 2 diabetes and NASH (Fig. 1D–F), as well as in the livers of mice induced by high-fat diet (HFD) alone or HFD combined with carbon tetrachloride (CCL4) treatment (Fig. 1G, H). There is a coherence in one NASH model established by Guo et al. [41]. Human LO2 cells or mouse AML12 cells were separately exposed to varying dose of cholesterol to establish cell models, considering the key role of NR5A2 as a transcription factor in cholesterol metabolism. Notably, at 80 μg/mL cholesterol concentration, LO2 cells exhibited a significant reduction in NR5A2 expression compared to AML12 cells which required 320 μg/mL for a similar effect (Fig. 1I).

### The emergence of spontaneous inflammation predated the formation of lipid droplets in the livers of hepatocyte-specific Nr5a2 knockout mice

The Nr5a2^HKO^ mice, a hepatocyte-specific knockout mice model of Nr5a2, was generated by crossbreeding Nr5a2^flox/flox^ mice with Alb-Cre mice (Fig. S2A, S2B). Mouse tail and liver DNA were extracted for successful verification of the knockout (Fig. S2C,S2D). Subsequently, the specific knockout of Nr5a2 in hepatocytes rather than other tissues was further confirmed (Fig. S2E–S2I).

**Fig. 2:**
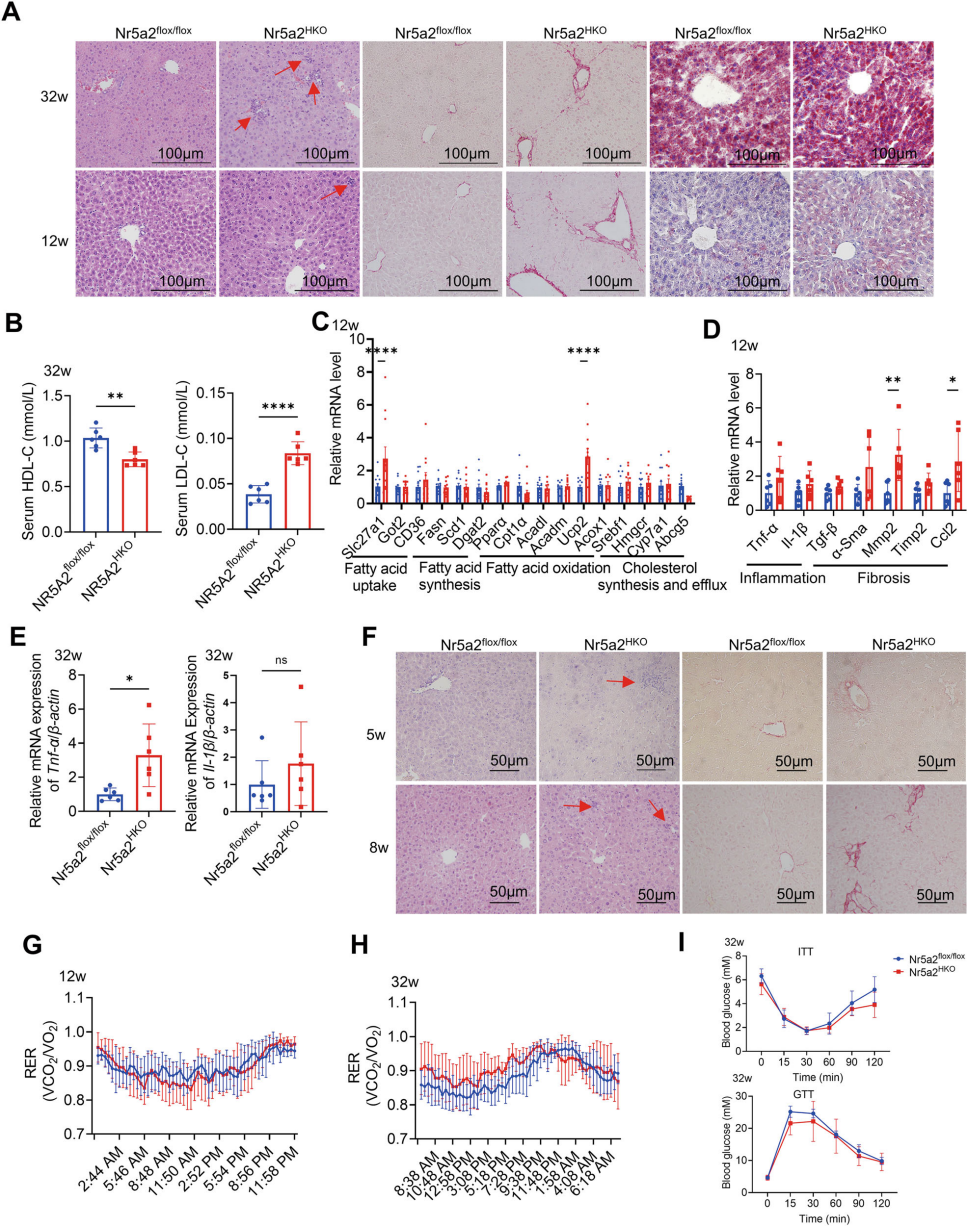
The hepatic inflammation and fibrosis preceded lipid accumulation in Nr5a2^HKO^ mice. **A** Liver morphology of 32-week-old and 12-week-old Nr5a2^HKO^ mice and their littermate was examined. **B** Serum levels of LDL cholesterol and HDL cholesterol were measured in the 32-week-old Nr5a2^HKO^ mice model. *N* = 6/group, **P < 0.01, ****P < 0.0001. Data are presented as means ± SD. **C**, **D** RT-qPCR analysis was performed to assess the expression of genes related to lipid metabolism (**C**) (2 technical replicates), as well as inflammation and fibrosis (**D**), in the liver of 12-week-old Nr5a2^HKO^ mice model. *N* = 6/group, *P < 0.05, **P < 0.01, ****P < 0.0001. Data are presented as means ± SD. **E** The expression levels of *Tnf-α* and *Il-1β* were determined by RT-qPCR in the liver of 32-week-old Nr5a2^HKO^ mice model. *N* = 6/group, ns, not significant, *P < 0.05. Data are presented as means ± SD. **F** Liver paraffin sections from 5-week-old and 8-week-old Nr5a2^HKO^ mice model were stained with HE and sirius red for histological examination. **G**, **H** Respiratory exchange ratio was measured in the 12-week-old (**G**) and 32-week-old (**H**) Nr5a2^HKO^ mice model. *N* = 6/group, data are presented as means ± SD. **I** Insulin tolerance test (ITT) and glucose tolerance test (GTT) were conducted on the 32-week-old Nr5a2^HKO^ mice model. *N* = 6/group, data are presented as means ± SD.

The 12-week-old and 32-week-old Nr5a2^HKO^ mouse models were cultivated. No discernible differences in the physical appearance and weight of the body, liver, and adipose tissue were observed between Nr5a2^flox/flox^ and Nr5a2^HKO^ mice at 32 weeks of age (Fig. S3A–S3E). Liver morphology analysis revealed exacerbated lipid accumulation, inflammation, and fibrosis in the 32-week-old Nr5a2^HKO^ mice compared to their littermates, consistent with reduced serum HDL-cholesterol levels and increased LDL-cholesterol levels (Fig. 2A, B). The presence of inflammatory infiltrate and fibrosis in the paraffin liver sections of Nr5a2^HKO^ mice at 12 weeks of age is noteworthy due to the absence of visible lipid droplets formation (Fig. 2A).

Liver lipid-related genes has an inconsistent tendency in the Nr5a2^HKO^ mice model (Fig. 2C). The expression of inflammation- and fibrosis-related genes was found to be upregulated in the liver of Nr5a2^HKO^ mice, without any compensatory effects (Fig. 2D, E). Liver morphology analysis in 5-week-old and 8-week-old Nr5a2^HKO^ mouse models revealed early onset of inflammation infiltration and fibrogenesis in the liver (Fig. 2F). At the age of 12 weeks, there were no significant differences observed in the respiratory exchange ratio (RER) between Nr5a2^flox/flox^ and Nr5a2^HKO^ mice (Fig. 2G). However, in 32-week-old Nr5a2^HKO^ mice, there was an upward trend observed, suggesting a disruption in lipid oxidation within the Nr5a2^HKO^ group (Fig. 2H). The activity and heat production were comparable between these two groups; however, Nr5a2^HKO^ mice exhibited a prolonged duration of rest state (Fig. S3F–S3I). In the 32-week model, change of serum triacylglycerol indicated an upregulation of lipid transport into liver (Fig. S3J, S3K). ALT and AST provided valueless information in 32-week Nr5a2^HKO^ mice model (Fig. S3L, S3M). ITT and GTT tests demonstrated that glucose metabolism remained unaffected in the Nr5a2^HKO^ group (Fig. 2I). These findings suggest that hepatocyte-specific knockout of Nr5a2 initially leads to inflammation followed by lipid accumulation and accelerated NASH progression independent of glucose metabolism. However, the underlying mechanism for this phenomenon remains unclear.

### Pyroptosis was observed in the NR5A2-deficient cell lines and in hepatocyte-specific Nr5a2 knockout mice

**Fig. 3:**
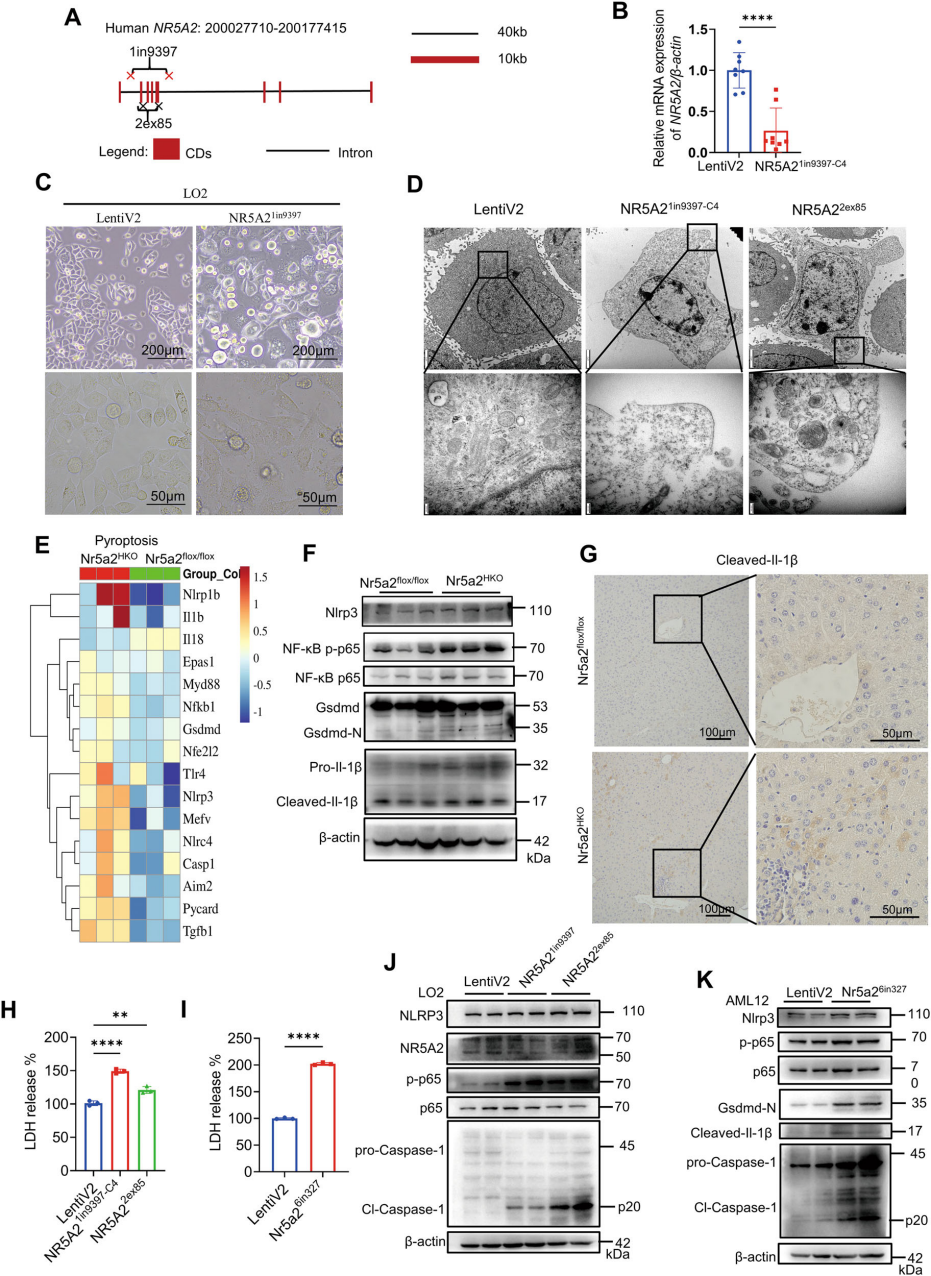
Pyroptosis was observed in the hepatocytes of Nr5a2^HKO^ mice, as evidenced by the occurrence of cell death in NR5A2-deficient cell lines. **A** A schematic diagram illustrating the design of *NR5A2* knockout in LO2 cell line. **B** RT-qPCR analysis was performed to determine the expression level of *NR5A2* in the LO2-NR5A2^1in9397-C4^ cell line. LentiV2 refer to the LentiCRISPR-v2 vector. *N* = 8/group, ****P < 0.0001. Data are presented as means ± SD. **C** Morphological changes associated with cell death were observed using optical microscopy during the monoclonal cell line cultivation. **D** Morphological changes associated with cell death were observed using transmission electron microscope (TEM) in the LO2-LentiV2 (control group), LO2-NR5A2^1in9397-C4^ and LO2-NR5A2^2*ex*85^ cell lines, Scale bars, 2 μm and 200 nm. **E** Heatmap representation of genes related to pyroptosis based on RNA-seq data obtained from 5-week-old Nr5a2^HKO^ mice model, *N* = 3/group. **F** Western blotting was conducted to assess the expression levels of pyroptosis molecules in the liver tissue of 5-week-old Nr5a2^HKO^ mice model. **G** Immunohistochemical staining of Cleaved-Il-1β in the liver paraffin sections of 12-week-old Nr5a2^HKO^ mice model. **H**, **I** Lactic dehydrogenase (LDH) release of LO2-NR5A2^1in9397-C4^ (**H**), LO2-NR5A2^2*ex*85^ (**H**), AML12-Nr5a2^6in327^ (**I**) and their control cell lines. *N* = 3/group, **P < 0.01, ****P < 0.0001. Data are presented as means ± SD. **J**, **K** Western blotting analysis was performed to evaluate key molecules involved in the pyroptosis process in the LO2-NR5A2^1in9397^ (**J**), LO2-NR5A2^2*ex*85^ (**J**) and AML12-Nr5a2^6in327^ (**K**) cell lines, respectively.

To investigate the functional role of NR5A2, we employed a gene knockout strategy in cell lines. For subsequent reintroduction of NR5A2 different isoforms, specifically, we designed two guide RNAs (gRNAs) targeting intron 1 and intron 5 to excise NR5A2 from LO2 cell line, referred to as LO2-NR5A2^1in9397^ (Fig. 3A). Additionally, primers flanking the cleavage sites were designed for verification of exon2 to exon5 removal. Agarose gel electrophoresis revealed a distinct band at 521 bp band following excision, while no amplification was observed in the control group due to the sequence length constraints (Fig. S4A). Two gRNAs targeting exon2 and exon5 were selected to enhance the efficiency of knockout in LO2 cell line, designated as LO2-NR5A2^2*ex*85^ (Fig. 3A). The expected band size indicating successful excision between the two cleavage sites should be approximately 202 bp (Fig. S4A). Surprisingly, complete knockout of NR5A2 was not achieved; however, a significant reduction in expression was observed (Fig. S4B). Replication of the experiment confirmed that one monoclonal cell line, LO2-NR5A2^1in9397-C4^, exhibited stable haploid expression of NR5A2 (Figs. 3B, S4C and S4D). The attempt to culture LO2-NR5A2^2*ex*85^ monoclonal cell line was unsuccessful due to the lethal consequences of NR5A2 loss [40]. In the AML12 cell line, the knockout strategy for Nr5a2 and the identification of Nr5a2 expression were conducted similarly as in the LO2-NR5A2^1in9397^ cell line (Fig. S4E, S4F). This interfered cell line was designated as AML12-Nr5a2^6in327^, which could not be maintained as a monoclonal cell line. During the process of monoclonal cell culture, we observed a distinct form of cell death that differed from puromycin-induced cell death (Fig. 3C). Considering the significant inflammatory phenotype exhibited by NR5A2^HKO^ mice, we hypothesized that this novel form of cell death may represent pyroptosis. Transmission electron microscope (TEM) displayed a consistent tendency of LO2-NR5A2^1in9397-C4^ and LO2-NR5A2^2*ex*85^ cell lines: decreased electron density of the cell matrix, mitophagosomes appearance and cell membrane swelling (Fig. 3D).

Liver RNA-Seq was conducted on 5-week-old Nr5a2^HKO^ mice and their littermates at LC-Bio Technologies (HangZhou) Co.,Ltd. Heatmaps depicting the clustering of pyroptosis, ferroptosis and cuproptosis pathways were generated for both Nr5a2^HKO^ mice and their littermate (Figs. 3E and S4G). The results revealed an activation of the pyroptosis pathway in Nr5a2^HKO^ mice, while the ferroptosis and cuproptosis pathways remained unaffected. This hypothesis was further supported by the detection of Gsdmd-N, a key marker of pyroptosis, in liver tissue from 5-week-old Nr5a2^HKO^ mice (Fig. 3F). The phosphorylation of NF-κB-p65 was enhanced, accompanied by the upregulation of Nlrp3 and the cleavage of IL-1β (Fig. 3F, G). The lactic dehydrogenase (LDH) in the supernatant of NR5A2 deficiency liver cell lines presented an upregulated tendency (Fig. 3H, I). In LO2 cell lines, both NR5A2^1in9397^ and NR5A2^2*ex*85^ exhibited cleaved Caspase-1, a typical molecule responsible for GSDMD cleavage (Fig. 3J). The AML12-Nr5a2^6in327^ cell line showed a corresponding trend in this death pathway, demonstrating evident Gsdmd-N expression (Fig. 3K). Immunohistochemical staining of Caspase-1 in the livers of Nr5a2^flox/flox^ and Nr5a2^HKO^ mice supported our hypothesis that Nr5a2 knockout induces pyroptosis in hepatocytes (Fig. S4H). We observed an upregulation of pro-Caspase-1 in the AML12-Nr5a2^6in327^ cell line (Fig. 3K), which was further confirmed by immunofluorescence analysis (Fig. S4I). Additionally, mRNA levels of NLRP3 were enhanced in NR5A2-deficient cell lines (Fig. S4J,S4K). Human IL-1β in the supernatant of LO2-NR5A2^1in9397-C4^ and LO2-NR5A2^2*ex*85^ cell lines were increased (Fig. S4L).

### Pyroptosis in NR5A2-knockout cell lines can be attributed to ROS-induced activation of NF-κB pathway

**Fig. 4:**
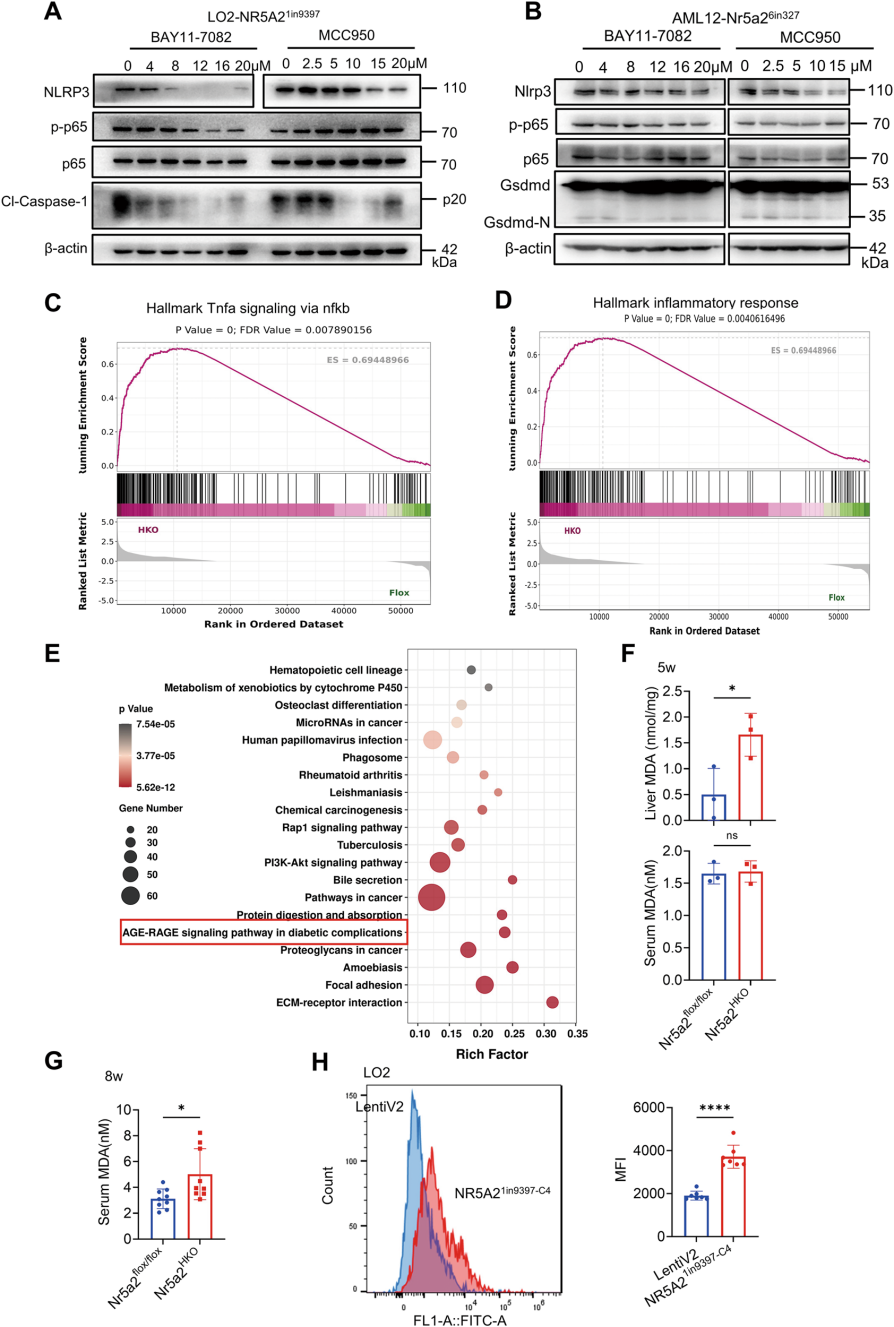
ROS serves as a pivotal factor in pyroptosis activation through NF-κB pathway in the context of NR5A2 deficiency. **A**, **B** Western blotting analysis was performed to detect the expression of pyroptosis-related molecules in the LO2-NR5A2^1in9397^ (**A**) and AML12- Nr5a2^6in327^ (**B**) cell lines after treated with gradient concentrations of inhibitors BAY11-7082 and MCC950. **C**, **D** GSEA was conducted to analyze the NF-κB pathway (**C**) and inflammation (**D**) using RNA-seq data from 5-week-old Nr5a2^HKO^ mice model. **E** KEGG Enrichment Scatterplot of 1285 different expressed genes (DEGs), based on the RNA-seq data from 5-week-old Nr5a2^HKO^ mice model. **F** MDA content assay was performed to measure liver and serum levels in the 5-week-old Nr5a2^HKO^ mice model. *N* = 3/group, ns, not significant, *P < 0.05. Data are presented as means ± SD. **G** Serum MDA content assay of 8-week-old Nr5a2^HKO^ mice model (3 technical replicates). *N* = 3/group, *P < 0.05. Data are presented as means ± SD. **H** ROS assay was carried out in the LO2-NR5A2^1in9397-C4^ cell line and its negative control. *N* = 7/group, ****P < 0.0001. Data are presented as means ± SD.

The NF-κB pathway, which is upstream of pyroptosis, plays a prominent role in the NOD-like receptor signaling pathway. Various inhibitors targeting the NF-κB pathway were utilized to elucidate its precise regulatory mechanism. It was observed that increasing concentrations of BAY11-7082, a well-known inhibitor of IκBα phosphorylation, led to a gradual decrease in Caspase-1 cleavage in the LO2-NR5A2^1in9397^ cell line (Fig. 4A). MCC950, an effective and selective inhibitor of NLRP3, does not interfere with NF-κB-p65 phosphorylation. The concentration-dependent decrease in cleaved Caspase-1 was evident upon treatment with increasing concentrations of MCC950 (Fig. 4A). The inhibition of Gsdmd-N production by BAY11-7082 and MCC950 in the AML12-Nr5a2^6in327^ cell line confirmed that the effect of Nr5a2 deletion on pyroptosis is not altered due to species differences (Fig. 4B). The mildly intensified phosphorylation of the NF-κB-p65 upstream moleculars observed in the liver of Nr5a2^HKO^ mice model provided further support for our assumptions (Fig. S5A). Therefore, NR5A2 knockout in mice or liver cell lines activated pyroptosis via the NF-κB-NLRP3 pathway. Gene Set Enrichment Analysis (GSEA) revealed upregulation of inflammation hallmarks associated with the NF-κB pathway in Nr5a2^HKO^ mice (Fig. 4C, D). However, there was no increased expression of TLR2 and MyD88, classical components in the upstream NF-κB pathway, in LO2-NR5A2^1in9397^ and LO2-NR5A2^2*ex*85^ cell lines compared to the negative control (Fig. S5B).

To investigate the activation of the NF-κB pathway, we performed Kyoto Encyclopedia of Genes and Genomes (KEGG) pathway enrichment analysis using liver RNA-Seq data from 5-week-old Nr5a2^HKO^ mice model. Notably, the Age-Rage signaling pathway in diabetic complications emerged as one of the top five candidate pathways, characterized by upregulated ROS levels (Figs. 4E and S5C). Additionally, MDA, a marker for ROS production, was detected in both Nr5a2^flox/flox^ and Nr5a2^HKO^ mouse livers. At the age of 5 weeks, a significant elevation in hepatic MDA levels was observed in Nr5a2^HKO^ mice, while no such increase was detected in the serum (Fig. 4F). By 8 weeks, Nr5a2^HKO^ mice exhibited a noticeable increase in serum MDA levels (Fig. 4G). Consistently, ROS levels were significantly elevated in the LO2-NR5A2^1in9397-C4^ cell line, mirroring the findings observed in Nr5a2^HKO^ mice (Fig. 4H).

### Downregulation of ALDH1B1 in Nr5a2^HKO^ mice exacerbates ROS level and activates the NF-κB pathway

**Fig. 5:**
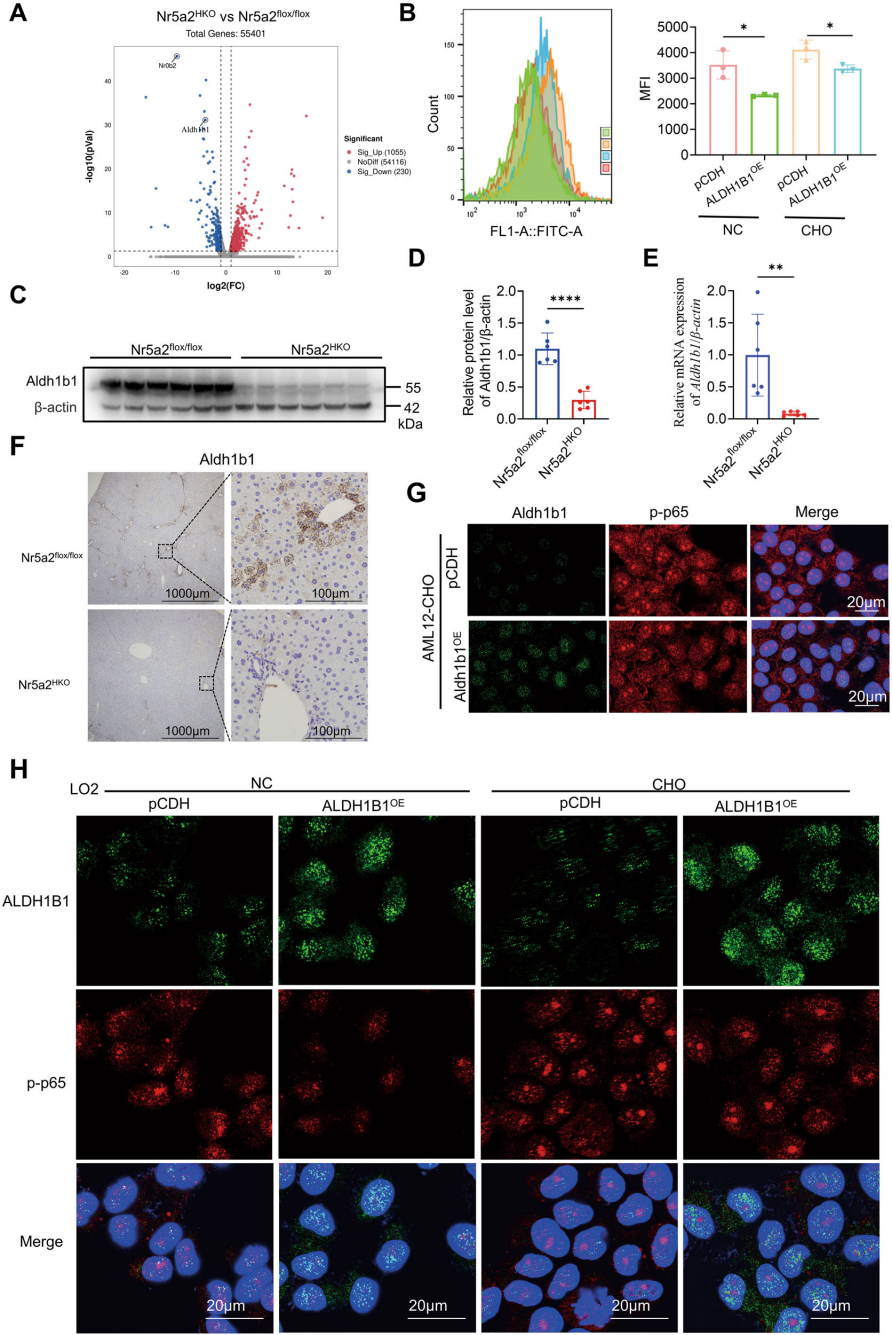
ALDH1B1 exhibited downregulation in the liver of Nr5a2^HKO^ mice and was found to modulate the NF-κB signaling pathway. **A** Volcano plot. The plot displayed -log10(P value) versus log2(fold change) of 1285 differentially expressed genes (DEGs), with 1055 genes upregulated and 230 genes downregulated in the liver of 5-week-old Nr5a2^HKO^ mice compared to their littermates. **B** ROS assay of LO2 cell line overexpressing ALDH1B1. CHO: cholesterol. Cholesterol was used at a concentration of 80 μg/mL for 24 h. pCDH refers to pCDH_EF1a-IRES-puro_BsrG1 vector. *N* = 3/group, *P < 0.05. Data are presented as means ± SD. **C**–**F** Western blotting (**C**), its statistical analysis (**D**), RT-qPCR assay (**E**), and immunohistochemical staining (**F**) were performed to evaluate Aldh1b1 expression in the liver of 12-week-old Nr5a2^HKO^ mice model. *N* = 6/group, **P < 0.01, ****P < 0.0001. Data are presented as means ± SD. **G**, **H** Immunofluorescence staining was conducted to examine ALDH1B1 and phosphorylated NF-κB p65 levels in the AML12 (**G**) and LO2 (**H**) cell lines overexpressing ALDH1B1. Cholesterol was used at a concentration of 80 μg/mL for 24 h in the LO2 cell line, while it was used at a concentration of 320 μg/mL for 24 h in the AML12 cell line.

Aldh1b1, exhibiting a higher oxidation efficiency in MDA compared to 4-HNE, emerged as one of the significantly altered genes in the Nr5a2^HKO^ mice according to the volcano plot (Figs. 5A and S6A) [42]. Given that Aldh2 demonstrates resistance to oxidative stress, it is reasonable to hypothesize that Aldh1b1 may function similarly [43]. There was a significant downregulation of ROS when ALDH1B1 was overexpressed in the LO2 cell line, both in the absence and presence of cholesterol (Fig. 5B). Although one molecular, Sod2, has been implicated in ROS changes in hepatocytes stimulated with the NR5A2 agonist, no regulation was observed in Nr5a2^HKO^ mice model in our study [44].

The liver of Nr5a2^HKO^ mice exhibited a significant reduction in Aldh1b1 expression (Fig. 5C–F). Subsequently, we investigated the impact of ALDH1B1 on the NF-κB pathway. Upon cholesterol stimulation, an attenuated immunofluorescence signal for phosphorylated NF-κB-p65 in the nucleus was observed in the Aldh1b1-overexpressing AML12 cell line, indicating that Aldh1b1 inhibited the activation of the NF-κB pathway (Fig. 5G). The observed alterations in the LO2 cell line consistently yielded significant results (Figs. 5H and S6B). Subsequent knockout of ALDH1B1 in the LO2 cells led to activation of the NF-κB pathway, accompanied by upregulation of its downstream molecule NLRP3 (Fig. S6C–S6E). Additional cholesterol stimulation, which exacerbates ROS production, can accentuate the observed effects (Figs. 5H and S6D) [45].

### NR5A2 transcriptionally upregulates ALDH1B1 expression by directly binding to its promoter region in both mouse and human

**Fig. 6:**
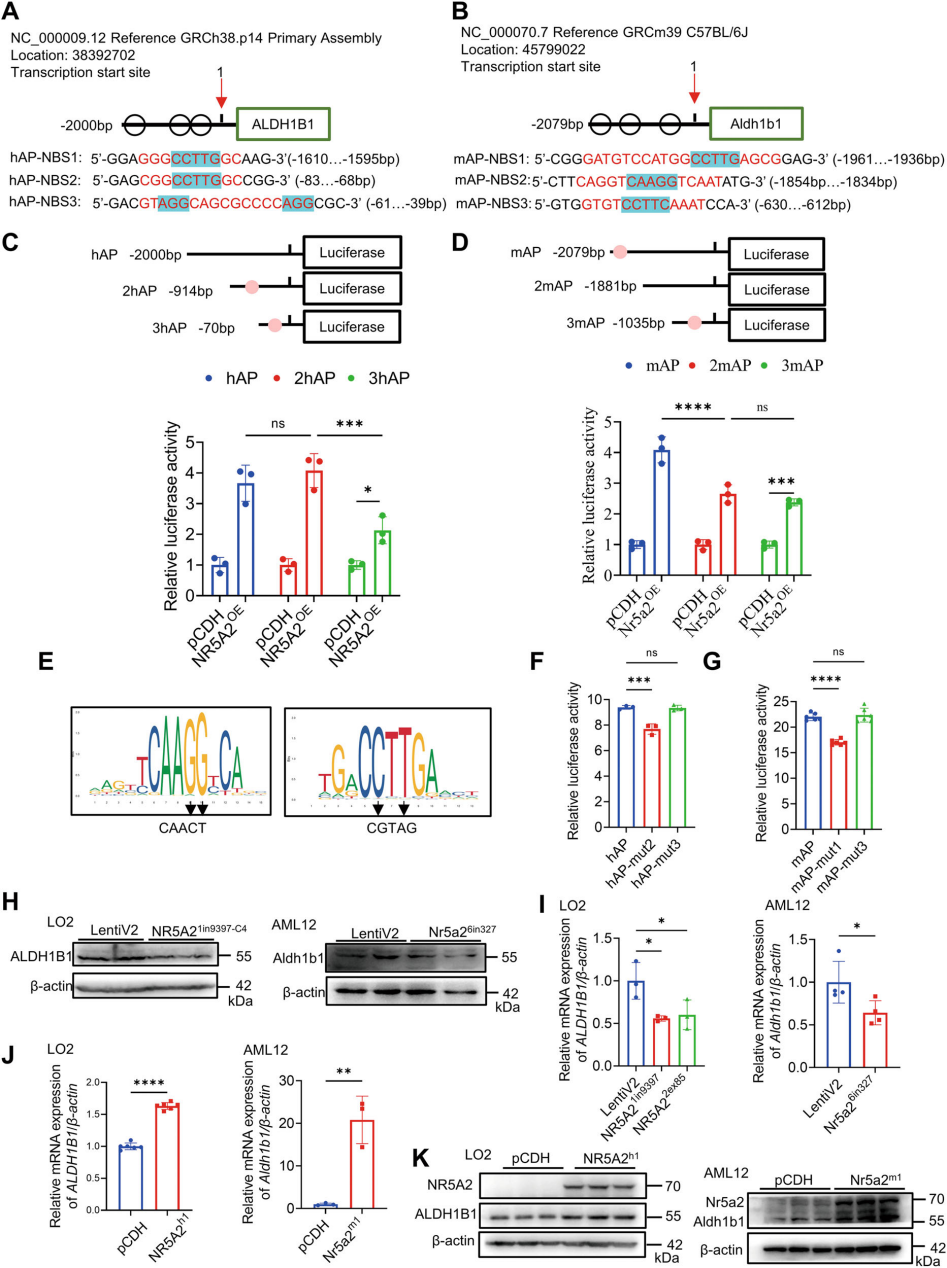
NR5A2 transcriptionally upregulates the expression of ALDH1B1. **A**, **B** Schematic representation of predicted NR5A2 binding sites in the promoter region of human (**A**) and mouse (**B**) *ALDH1B1*, as assessed by the dual luciferase reporter assay. The promoter region spans from −2000 bp to +100 bp relative to the transcriptional start site in human and from −2079 bp to +100 bp in mice. h(m)AP-NBS: NR5A2 binding site within the promoter region of human (mouse) *ALDH1B1*. (C-D) Evaluation of NR5A2 transcription activity on truncated versions of human (**C**) and mouse (**D**) *ALDH1B1* promoter regions using a dual-luciferase reporter system. h(m)AP: promoter region of human (mouse) *ALDH1B1*. 2/3 h(m)AP: The second or third truncation of the promoter region. *N* = 3/group, ns, not significant, *P < 0.05, ***P < 0.001, ****P < 0.0001. Data are presented as means ± SD. **E** Schematic representation of NR5A2 binding site mutations in the promoter region of *ALDH1B1*. **F**, **G** Dual-luciferase reporter system employed to analyze the transcriptional activity of NR5A2 on the mutated human (**F**) and mouse (**G**) *ALDH1B1* promoter region. h(m)AP-mut1/2/3 indicates mutation at the first, second or third predicted binding site was mutant in human (mice). Human: *N* = 3/group, mouse: *N* = 6/group, ns not significant, ***P < 0.001, ****P < 0.0001. Data are presented as means ± SD. **H** Western blotting analysis of ALDH1B1 expression in the LO2-NR5A2^1in9397-C4^ and AML12-Nr5a2^6in327^ cell lines. **I** RT-qPCR quantification of *ALDH1B1* expression in the LO2-NR5A2^1in9397^, LO2-NR5A2^2*ex*85^ and AML12-Nr5a2^6in327^ cell lines. LO2: *N* = 3/group, AML12: *N* = 4/group, *P < 0.05. Data are presented as means ± SD. **J**, **K** RT-qPCR assessment (**J**) and western blotting analysis (**K**) of ALDH1B1 expression in the LO2 and AML12 cell lines overexpressing NR5A2 isoform1. LO2: *N* = 6/group, AML12: *N* = 3/group, **P < 0.01, ****P < 0.0001. Data are presented as means ± SD.

After transfecting the LO2 cell line with various transcripts of *NR5A2* derived from different species, all isoforms of NR5A2 demonstrated an upregulation of ALDH1B1 expression (Fig. S6F). The sequence spanning from −2000 bp to +100 bp was submitted to both the Animal Transcription Factor Database (Animal TFDB3.0) and JASPAR database for precise prediction of NR5A2 binding sites. In the human *ALDH1B1* promoter region (hAP-NBS), three putative binding sites for human NR5A2 were identified at positions −1610 bp to −1595bp, −83 bp to −68 bp, and −61 bp to −39 bp, respectively (Fig. 6A). Similarly, the mouse *Aldh1b1* promoter area also harbored three predicted binding sites for mouse Nr5a2 at positions-1961 bp to −1936 bp, −1854 bp to −1834 bp, and −630 bp to −612 bp (Fig. 6B). Luciferase activity was enhanced upon the addition of the corresponding *ALDH1B1* promoter in response to human and mouse NR5A2 overexpression (Fig. S6G). Notably, the fluorescence intensity of the half truncation variant, 2hAP, remained comparable to that of the full sequence, suggesting that either the first predicted binding site or the deleted sequence is not influenced by NR5A2 regulation (Fig. 6C). After eliminating the second predicted binding site, the fluorescence intensity of 3hAP was significantly attenuated (Fig. 6C). The dual luciferase reporter assay on truncations identified two potential binding sites, hAP-NBS2 and hAP-NBS3, within the promoter region of *ALDH1B1* for human NR5A2 (Fig. 6C). Similarly, analysis of Nr5a2 regulation on *Aldh1b1* in mice revealed mAP-NBS1 and mAP-NBS3 as candidate binding sites (Fig. 6D).

According to the JASPAR database, NR5A2 binds to the consensus sequence 5’-CAAGG-3’ or 5’-CCTTG-3’, which is consistent with previous research findings (Fig. 6E) [46, 47]. The selection of mutant site was based on the binding frequency of each base. Specifically, 5’-CAAGG-3’ and 5’-CCTTG-3’ were mutated to 5’-CAACT-3’ and 5’-CGTAG-3’, respectively (Fig. 6E). The luciferase activity assay confirmed the accuracy of hAP-NBS2 prediction, while no significant difference was observed for the third mutation compared to the original sequence (Fig. 6F). Mouse Nr5a2 exhibited successfully binding to mAP-NBS1 but failed to bind mAP-NBS3 (Fig. 6G).

ALDH1B1 expression was significantly attenuated in the LO2-NR5A2^1in9397-C4^ and AML12-Nr5a2^6in327^ cell lines (Fig. 6H, I). Lentiviral-mediated overexpression of the full-length isoform of human NR5A2 in LO2 or mouse Nr5a2 in AML12 resulted in augmented transcript and protein levels of both human and mouse ALDH1B1 (Fig. 6J, K).

### ALDH1B1 attenuated the pyroptosis in NR5A2-deficient cell lines, and diverse isoforms of NR5A2 demonstrated a comparable effect

**Fig. 7:**
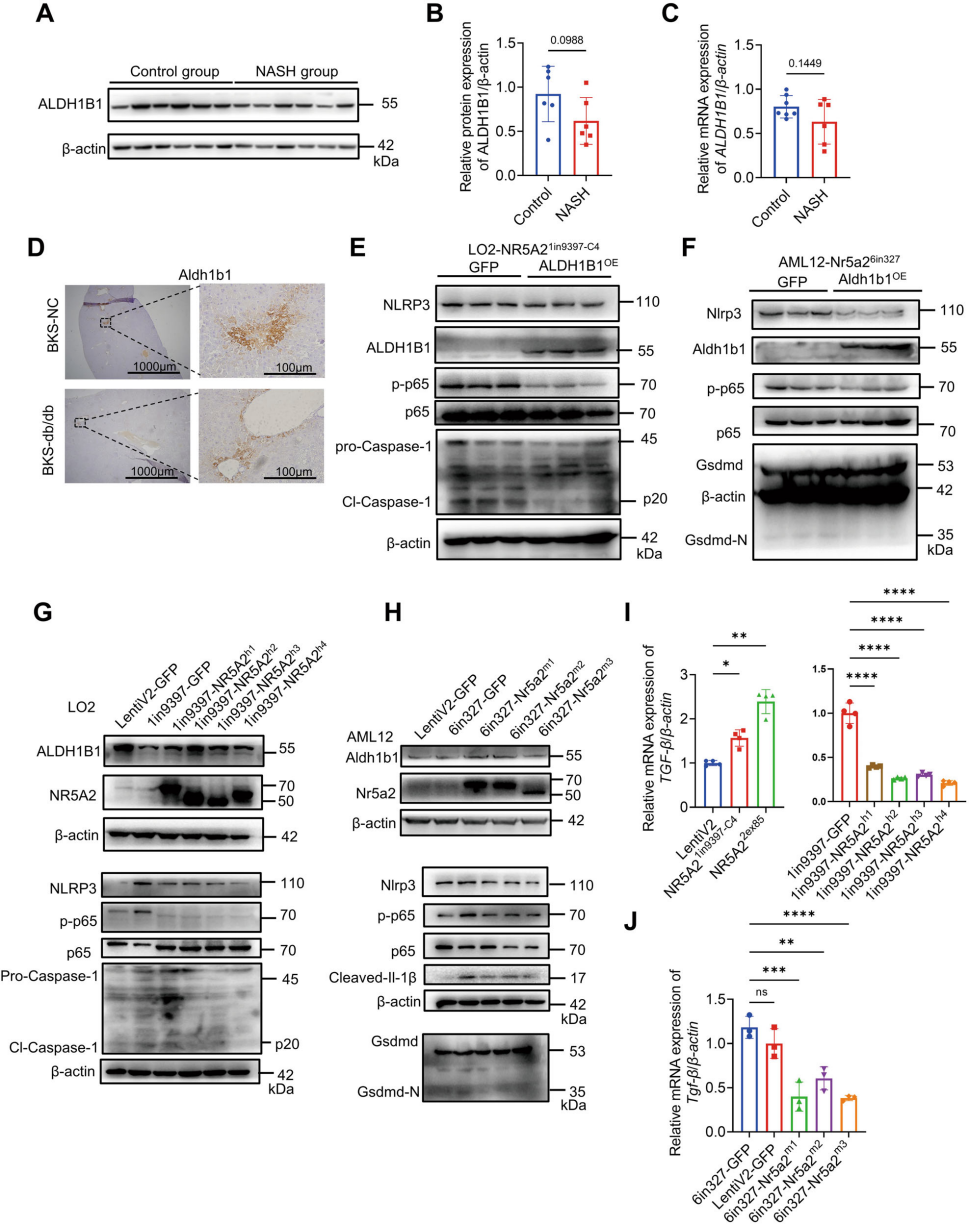
ALDH1B1 can ameliorate the activation of pyroptosis, and NR5A2 different isoforms exhibit a similar function. **A**, **B** Western blotting analysis was performed to detect the expression of ALDH1B1 in the livers of NASH patients and control group (**A**), followed by statistical analysis (**B**). *N* = 6/group, data are presented as means ± SD. **C** The mRNA level of *ALDH1B1* was measured in the livers of NASH patients and control group. *N* = 6/group, data are presented as means ± SD. **D** Immunohistochemistry staining was conducted to examine the expression of Aldh1b1 in the liver paraffin sections of BKS-db mice and BKS-NC mice. **E**, **F** The protein expression of pyroptosis molecules were assessed after overexpressing ALDH1B1 in the NR5A2^1in9397-C4^ LO2 cell line (**E**) and AML12-Nr5a2^6in327^ cell line (**F**). **G**, **H** The western blotting analysis was performed to evaluate the expression levels of ALDH1B1 and pyroptosis molecules after replenishment with different isoforms of NR5A2 in the LO2-NR5A2^1in9397-C4^ cell line (**G**) and AML12-Nr5a2^6in327^ cell line (**H**). **I**, **J** RT-qPCR was conducted to measure *TGF-β* mRNA expression in the LO2 cell line (**I**) and AML12 cell line (**J**), following haploid knockout or replenishment of NR5A2 with different isoforms. LO2: *N* = 4/group, AML12: *N* = 3/group, ns not significant, *P < 0.05, **P < 0.01, ***P < 0.001, ****P < 0.0001. Data are presented as means ± SD.

In the NASH group, ALDH1B1 expression was relatively lower in the human liver (Fig. 7A–C). Although Aldh1b1 expression in liver paraffin sections of BKS genetic mice was lower compared to C57BL/6J genetic mice, the distribution of Aldh1b1 was still wider in non-leptin knockout mice than in BKS-db mice (Fig. 7D). These results showed a similar trend to NR5A2 expression in the NASH model. We overexpressed ALDH1B1 in the LO2-NR5A2^1in9397-C4^ and AML12-Nr5a2^6in327^ cell lines, resulting in its effective inhibition of the pyroptosis pathway (Fig. 7E, F). However, no cell death was observed upon ALDH1B1 knockout in the LO2 cell line. Therefore, the principal cause of pyroptosis in NR5A2-deficient cells is ascribed to the accumulation of ROS due to compensatory overloaded lipid metabolism and the loss of reactive oxygen scavenging genes.

The overexpression of diverse isoforms of NR5A2 in the LO2-NR5A2^1in9397-C4^ cell line resulted in upregulation of ALDH1B1 and obvious inhibition of the NF-κB pathway (Fig. 7G). Similarly, the mouse Nr5a2 isoforms in the AML12-Nr5a2^6in327^ cell line showed the same result (Fig. 7H). The cleavages of caspase-1 in the LO2 cell line and Gsdmd in the AML12 cell line were abolished (Fig. 7G, H). Furthermore, Nr5a2^HKO^ mice exhibited distinct liver fibrosis compared to Nr5a2^flox/flox^ mice (Fig. 2A, F). TGF-β plays a pivotal role in regulating fibrosis and can be modulated by AP-1, a complex influenced by NR5A2 [48, 49]. Inhibition of NR5A2 in the LO2 and AML12 cell lines led to an upregulation of TGF-β, with all isoforms of NR5A2 exhibiting inhibitory regulatory effect (Fig. 7I, J). The moderately increased level of TGF-β observed in the AML12-Nr5a2^6in327^ cell line may be attributed to compensatory mechanisms involving residual NR5A2. Collectively, these rescue experiments involving different isoforms of NR5A2 provided evidence for their conserved function in promoting transcription and inhibiting pyroptosis (Figs. 7G–J, S1A and S1B).

## Discussion

The excessive accumulation of lipid may indicate the initiation of NAFLD spectrum. However, short-term HFD feeding induces mild inflammation, suggesting that inflammation is a consequence of prolonged lipid stimulation [50]. Hepatocyte-specific knockout mice of NR5A2 resulted in liver inflammation prior to the appearance of lipid droplets, which is in step with that inflammation may precede steatosis in certain instances [51]. NR5A2 was downregulated in patients with NASH and mouse models of NASH. The intervention of NR5A2 gave an interference in the upstream of complex regulation network especially in inflammation. To the best of our knowledge, NR5A2 is commonly reported to activate inflammation through AP-1, a transcription factor [52]. However, rescue experiment involving ALDH1B1 revealed that pyroptosis is primarily attributed to ROS rather than AP-1 in NR5A2 deficiency cell lines. This suggests that the inflammation observed in the Nr5a2^HKO^ mice is influenced by multiple factors.

The activation of pyroptosis via NF-κB pathway was found to be dependent on ROS in NR5A2 knockout conditions. Apoptosis occurred in cell lines with inhibited NR5A2 expression [21, 53]. Moreover, NR5A2 exhibited a protective role against cellular stimuli and promoted cell proliferation, thereby acting as a crucial molecule in cancer cells [54, 55]. In the duration of protection, NR5A2 demonstrated a close association with mitochondria [56]. Our finding revealed the downregulation of ALDH1B1, a mitochondrial enzyme, in the absence of NR5A2. Although an effective inhibitor called IGUANA-1 has been identified for ALDH1B1, no activator of ALDH1B1 has been investigated thus far. Given its potential as a cancer marker, targeting ALDH1B1 could be a promising strategy to inhibit cancer progression. However, exploiting the tissue-specific distribution and characteristics of this metabolic enzyme may offer opportunities for effectively preventing chronic diseases.

The rescue experiments of different NR5A2 isoforms showed no discernible difference in their impact on the transcriptional function and regulation of pyroptosis. The conserved region of NR5A2, starting from its DNA-binding domain, suggests that the A/B domain is not essential for its regulatory function. This finding aligns with a previous study [6].

There remains a gap in our understanding the intercellular interactions required for the survival of hepatocytes lacking Nr5a2 in vivo. It is plausible that endocrine or paracrine signaling plays a role in protecting hepatocytes from death. Further experiments are warranted to elucidate the underlying mechanisms responsible for this protective effect.

## Methods

### Human liver sample

All samples used in this study were obtained from the NAFLD Research Center, Department of Liver Medicine, the First Affiliated Hospital of Wenzhou Medical University. Detailed information can be found in Table S1.

### Mouse model

Mice were housed and bred in a temperature-controlled and specific pathogen-free facility with 12-h light/dark cycles and received food and water ad libitum.

a) BKS-db mice: BKS-db mice with a BKS genetic background were procured from GemPharmatech Co., Ltd. and maintained on a standard diet. Liver samples were collected at 16 weeks of age (6 mice/group).

b) The high-fat diet (HFD) and CCL4-induced NASH mice model [57]: C57BL/6J mice were divided into three groups (8 mice/group). The Chow group was fed a regular diet throughout their entire lifespan. Mice in HFD group were subjected to a high-fat diet (Research Diet, D12492) for a continuous 14-week period, starting from the age of 8 weeks. This group received weekly intraperitoneal injections of 200 µL corn oil (Shanghai Aladdin Biochemical, C116025). The HFD+CCl4 group received a combination of HFD and weekly infusion of carbon tetrachloride (CCl4) (Chemical Reagent, GB/T688-2011 from Tianli). The CCl4 was dissolved in 200 μL corn oil at an injection concentration of 0.4 μg/g of mouse body weight.

c) Nr5a2^HKO^ mice model: Loxp-labled Nr5a2 mice (Nr5a2^flox/flox^) and albumin-Cre transgenic mice were obtained from the Shanghai Model Organisms Center, Inc. Hepatocyte-specific knockout mice of Nr5a2 (Nr5a2^HKO^) were achieved by crossbreeding Nr5a2^flox/flox^ mice with albumin-Cre transgenic mice. We established four mice models based on their age: 5-week-old mice (3 mice/group), 8-week-old mice (3 mice/group), 12-week-old mice (6 mice/group), and 32-week-old mice (6 mice/group). Male Nr5a2^HKO^ and their littermate control, Nr5a2^flox/flox^ mice, were fed a standard diet. Glucose tolerance tests (GTT) were conducted on the 12 and 32-week-old mouse models following a 16-h fasting period, while insulin tolerance tests (ITT) were performed after a 4-h fasting period prior to anatomical sampling two weeks. The respiratory exchange ratio, activity, and heat production of Nr5a2^HKO^ and Nr5a2^flox/flox^ mice at the age of 12 weeks or 32 weeks were monitored using TSE PhenoMaster animal monitoring system. Mice were anesthetized after a 12-h fasting at the ages of 5, 8, 12, and 32 weeks for liver and adipose tissues weighing followed by rapid freezing in liquid nitrogen. Serum triglyceride, cholesterol, ALT, AST, HDL-C and LDL-C were measured at the Forth Xi’an People’s Hospital.

### Cell culture

LO2, AML12 and HEK293T cell lines were cultured in the high glucose-Dulbecco’s modified Eagle’s medium (Sigma, D5796) supplemented with 10% fetal bovine serum (Every Green, 11011-8611) and 1% penicillin–streptomycin–gentamicin Solution (Solarbio, P1410) at 37 °C in a humidified atmosphere containing 5% CO_2_. Selection of overexpression cell lines or knockdown cell lines was performed using either 1 μg/mL puromycin (Shyuanye, R23002) or 800 μg/mL neomycin (Shyuanye, B65949). Subsequently, half of the screen concentration of antibiotics was used to maintain the gene-edited cells. LO2 cells were stimulated with cholesterol (Sigma-Aldrich, L4646) at a concentration of 80 μg/mL while AML12 cells were subjected to a fourfold higher concentration. Inhibitors of BAY11-7082 (MCE, HY-13453) and MCC950 (MCE, HY-12815) were used in a gradient concentration to evaluate the pyroptosis pathway.

### DNA identification of mouse tail and liver tissue

The DNA from mouse tail and liver tissues were extracted using TIANamp Genomic DNA Kit (TIANGEN, DP304). Polymerase chain reaction (PCR) was performed to amplify the target region between forward and reverse primers relying on 2 × Taq PCR MasterMix II (TIANGEN, KT211). Subsequently, the PCR product was subjected to agarose gel electrophoresis (Biowest, G10) with GoldView (ZHHC, NE006) staining and visualized under ultraviolet light. The primers are listed in Table S2.

### CRISPR-Cas9

The knockout of NR5A2 in the cell was achieved by transfecting lentiCRISPR-v2 vector containing dual guide RNA (dgRNA). The constructed plasmid was transfected into cell lines using SuperKine™ Lipo3.0 Efficient Transfection Reagent (Abbkine, BMU111-CN), and positive clones were selected using puromycin (Shyuanye, R23002). The guide RNAs are listed in Table S3.

### Lentivirus packaging

The rescue of NR5A2 and ALDH1B1 in the cell lines was achieved using a lentivirus system. During the lentivirus packaging process, HEK293T cells were utilized as a tool and transfected with lentiviral helper plasmids psPAX2 and pMD2.G, along with the expression pCDH vector carrying the target genes. After 72 h, the polybrene (Shyuanye, R32418) was added the supernatant at a concentration of 1 μL/mL to enhance lentivirus infection efficiency. The pCDH_EF1a-IRES-puro_BsrG1 vector was used to overexpress genes in the normal cell lines. In order to avoid interference from puromycin antibiotic present in lentiCRISPR-v2 vector, we selected the pCDH vector with neomycin-resistant characteristics (pCDH-CMV-MCS-EF1-Neo) in the rescue experiment. Transfection of HEK293T cells was performed using Lipo293F™ transfect reagent (Beyotime, C0518). The G418 (Shyuanye, B65949) was purchased for neomycin selection. The primers for clone are listed in Table S4 and the recombinant DNA are listed in Table S5.

### Dual luciferase reporter

The sequences ranging from -2000 bp to +100 bp of human and mouse ALDH1B1 promoter were respectively inserted into the pGL4.35 [luc2P-9XGAL4UAS-Hygro] vector. Lentiviral expression vectors pCDH_EF1a-IRES-puro_BsrG1 were used for cloning the coding sequences of human and mouse NR5A2, resulting in two overexpressed NR5A2 vectors. The renilla luciferase-containing pRL-TK vector served as a control for transfection efficiency. Equal amounts of luciferase-containing vectors were co-transfected with either the empty pCDH vector or the pCDH vector carrying the NR5A2 insertion into 293T cells. Luciferase activity was measured using Thermo Scientific™ Nunc™ F96 MicroWell™ (136101) plates according to the instructions of Dual-Luciferase Report kit (Promega, E1910).

### Western blotting

Proteins were extracted from cells or animal tissues using RIPA lysis buffer (ZHHC, PL001), supplemented with protease inhibitor cocktail (MCE, HY-K0010) and phenylmethylsulfonyl fluoride benzylsulfonyl fluoride (PMSF) (ZHHC, PL012) under ice bath conditions. Subsequently, the proteins (30 μg/sample) were subjected to 10% sodium dodecyl sulfate-polyacrylamide gel electrophoresis and transferred to polyvinylidene fluoride membranes (Millipore, IPVH00010, 0.45 µm). The primary antibody (1:1000) was incubated overnight at 4°C followed by incubation with the secondary antibody (1:2500) at room temperature for 1.5 h. The antibodies are listed in the Table S5. Finally, enhanced chemiluminescence (ECL) was performed using Omni-ECL™ Femto Light Chemiluminescence Kit (Epizyme, SQ201).

### Quantitative RT-PCR (RT-qPCR)

Total RNA was extracted from cells and tissues using TRIGene Reagent (GenStar, P118) according to the manufacturer’s instructions. Subsequently, cDNA synthesis was performed using a RevertAid Master Mix, with DNase I kit (Thermo Fisher Scientific, M16325). For quantitative real-time PCR analysis, 2×RealStar Fast SYBR qPCR Mix (GenStar, A301) was employed under the three-step PCR amplification procedure. The data was analyzed using the 2^^-ΔΔ^CT method. The primers for RT-qPCR are listed in Table S6.

### Hematoxylin-eosin staining (HE stain) and Sirius staining

Liver tissues were fixed in 4% paraformaldehyde for 48 h, followed by embedding in paraffin and sectioning into 4-μm thick slices. After deparaffinization, the paraffin sections were stained with hematoxylin and eosin, or Sirius red. Subsequently, the liver sections underwent dehydration using a gradient alcohol and xylene. Following sealing with neutral balsam, the morphology of liver sections was assessed and documented under an optical microscope.

### Immunohistochemical staining

The paraffin sections were subjected to citrate antigen retrieval and antigen blocking after deparaffinization. Primary antibodies were incubated overnight at 4 °C, followed by incubation with second antibodies at 37 °C for 1 h. The experimental procedures adhered to the manufacturer’s instructions of the Streptavidin Rabbit & Mouse HRP Kit (DAB) (Cwbio, CW2069). Antibodies used in immunohistochemical staining included NR5A2 (Everest, EB12283, 1:100), ALDH1B1 (Santa Cruz Biotechnology, SC-393583, 1:100), Caspase-1 (Proteintech, 22915-1-AP, 1:100) and cleaved-IL-1β (Affinity, AF4006, 1:100).

### Oil red O staining

The liver tissues in 4% paraformaldehyde solution were dehydrated using 20% sucrose and embedded in Neg-50 Frozen Section Medium (Thermo Fisher Scientific, 6502B). After thawing out from −20 °C, the liver frozen sections were allowed to equilibrate at room temperature for 10 min and then fixed with 4% paraformaldehyde for 15 min. Following a brief immersion in a solution of 60% isopropyl alcohol, the sections were stained with the oil red O staining solution for 10 min. A subsequent differentiation step using a solution of 60% isopropyl alcohol was performed to enhance interstitial clarity. Finally, hematoxylin was applied to label the nucleus.

### Immunofluorescence

The cells were fixed in 4% paraformaldehyde for 20 min and subsequently incubated in 0.1% triton for 10 min. Primary antibodies ALDH1B1 (Santa Cruz Biotechnology, SC-393583; 1:100) and Phospho-NF-κB p65 (Ser536) (Cell Signaling Technology, 93H1, #3033, 1:500) were co-incubated overnight at 4 °C. CoraLite488-conjugated Goat Anti-Mouse IgG(H + L) (Proteintech, SA00013-1, 1:100) and CoraLite594 – conjugated Goat Anti-Rabbit IgG(H + L) (Proteintech, SA00013-4, 1:100) were chosen as the second antibody. Antifade mounting medium (Coolaber, SL1841) containing DAPI was used to stain the nucleus and seal the slide. Confocal laser scanning microscope (Olympus FV3000) was employed for observation and data collection.

### Lactic dehydrogenase (LDH) release

The measurement of Lactic dehydrogenase (LDH) release was conducted using the LDH assay kit (Beyotime, C0016). Calculatoring formula: LDH release (%)=(treatment group-blank background)/(control group-blank background)*100.

### Transmission electron microscope (TEM)

The cells were centrifuged into a cell cluster and fixed in 2.5% glutaraldehyde fixative. The subsequent process was conducted as previously described [58]. Transmission electron microscopy (HITACHI, H-7650) was used to observe the morphology associated with cell death.

### Enzyme-linked immunosorbent assay (ELISA)

The ELISA was performed according to the instructions provided by the Human IL-1 beta ELISA Kit (Proteintech, KE00021).

### ROS measurement

The measurement of total reactive oxygen species (ROS) in cells was conducted using the DCFH-DA assay kit (Beyotime, S0033S). On account of the similar fluorescence spectra between DCF and FITC, the parameter settings for FITC were employed to detect DCF on the flow cytometer.

### MDA measurement

The MDA was detected according with the manufacturer’s instruction of an MDA detection kit (Beyotime, S0131).

### Quantification and statistical analysis

The Animal Research: Reporting of In Vivo Experiments (ARRIVE) guidelines were followed to instruct sample size design and to formulate inclusion and exclusion criteria. Group allocation was based on mouse genotype or human liver phenotype. Experimental operators were blinded when assessing the outcomes.

Statistical analysis was conducted using two-tailed unpaired Student’s *t* test and one- or two-way ANOVA in Prism 9 (GraphPad). The data were presented as mean ± SD. *P < 0.05, **P < 0.01, ***P < 0.001, ****P < 0.0001, ns, not significant.

## Supplementary information

Supplementary material

Original data

## Data Availability

All gene information used in this study can be accessed from the Gene datasets in the National Center for Biotechnology Information (NCBI) database (https://www.ncbi.nlm.nih.gov/gene). The datasets generated and analysed during the current study are available from the corresponding author on reasonable request.
