# Supplementary material for: Hepatocyte-specific NR5A2 deficiency induces pyroptosis and exacerbates non-alcoholic steatohepatitis by downregulating ALDH1B1 expression
Source: Cell Death Dis. 2024 Oct 23;15(10):770. doi: 10.1038/s41419-024-07151-1 (PMC11496806; doi:10.1038/s41419-024-07151-1)
Supplement: Supplementary file 1 — Supplementary material [file 41419_2024_7151_MOESM1_ESM.pdf]

Supplemental Information

Hepatocyte-specific NR5A2 Deficiency Induces Pyroptosis and Exacerbates Non-alcoholic Steatohepatitis by Downregulating ALDH1B1 Expression

Rong Zhao, Zizhen Guo, Kaikai Lu, Qian Chen, Farooq Riaz, Yimeng Zhou, Luyun Yang, Xiaona Cheng, Litao Wu, Kexin Cheng, Lina Feng, Sitong Liu, Xiaodan Wu, Minghua Zheng, Chunyan Yin, Dongmin Li

Figure S1

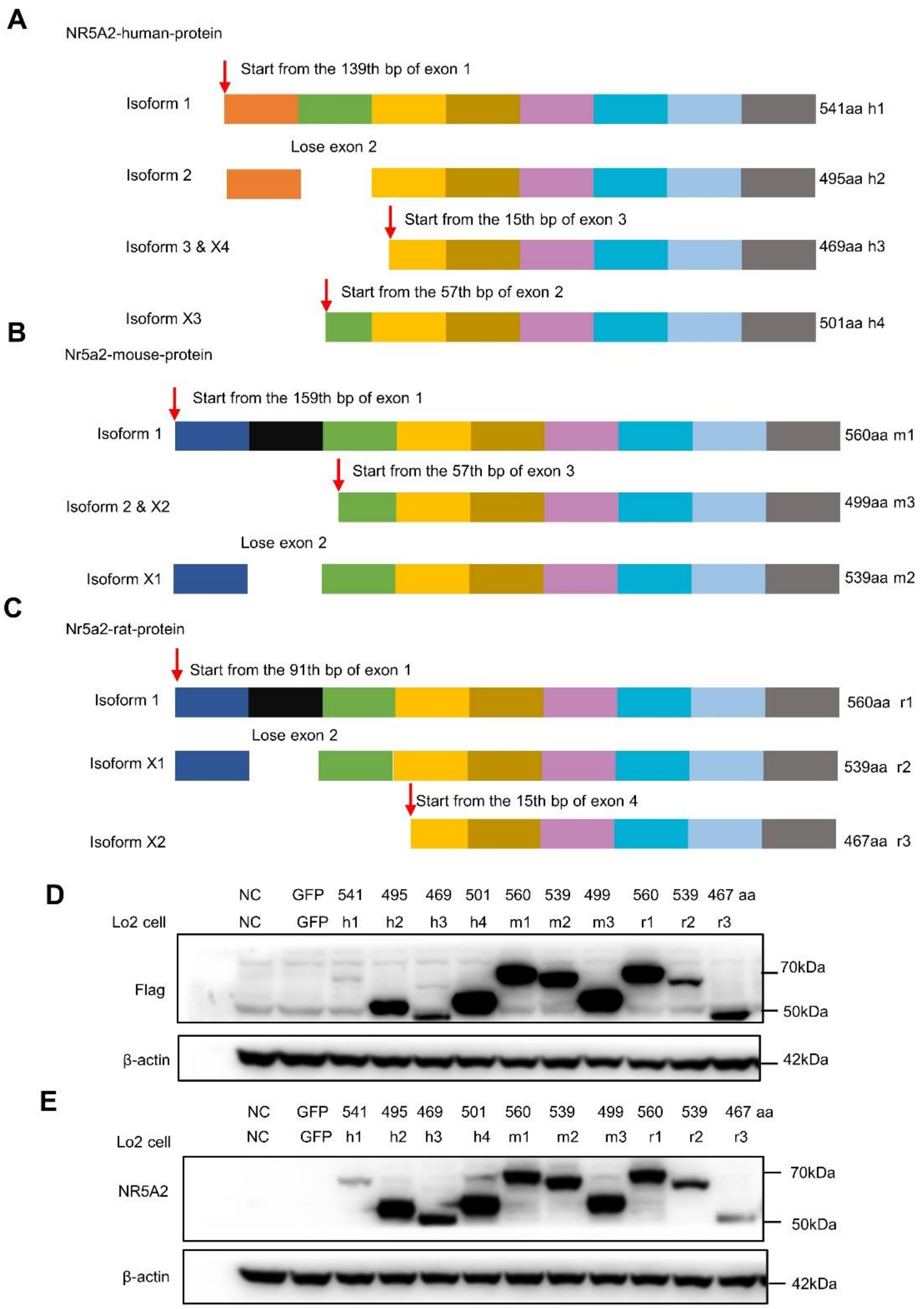

Figure S1. The schematic representation and validation of NR5A2 isoforms across different species.

(A-C) The schematic diagram of depicting distinct isoforms of NR5A2 across

various different species including human, mouse and rats. Different color rectangles represent individual exons, while identical colors across different species indicate a significant level of homology for these exons. The human NR5A2 isoforms encompass isoform 1, 2, 3&X4 (coding the same sequence) and X3. Isoform X1 and X2, new updated types, are not included in this study (A). Mouse Nr5a2 isoforms consist of isoform 1, 2 & X2(coding the same sequence), and X1 (B). Rat Nr5a2 isoforms include isoform 1, X1 and X2 (C). (D-E) Western blotting was conducted to detect different NR5A2 isoforms tagged with a flag sequence on the N-terminal using flag antibody (D）or NR5A2 antibody (E). The coding domain sequence (CDS) of NR5A2 different isoforms were cloned into pCDH_EF1a-IRES-puro_BsrG1 vector and transfected into LO2 cell line. After 36 hours, the protein collection and analysis were performed. Each lane is annotated with the amino acid numbers of NR5A2 different isoforms and serial number corresponding to Figure S1A-S1C.

Figure S2

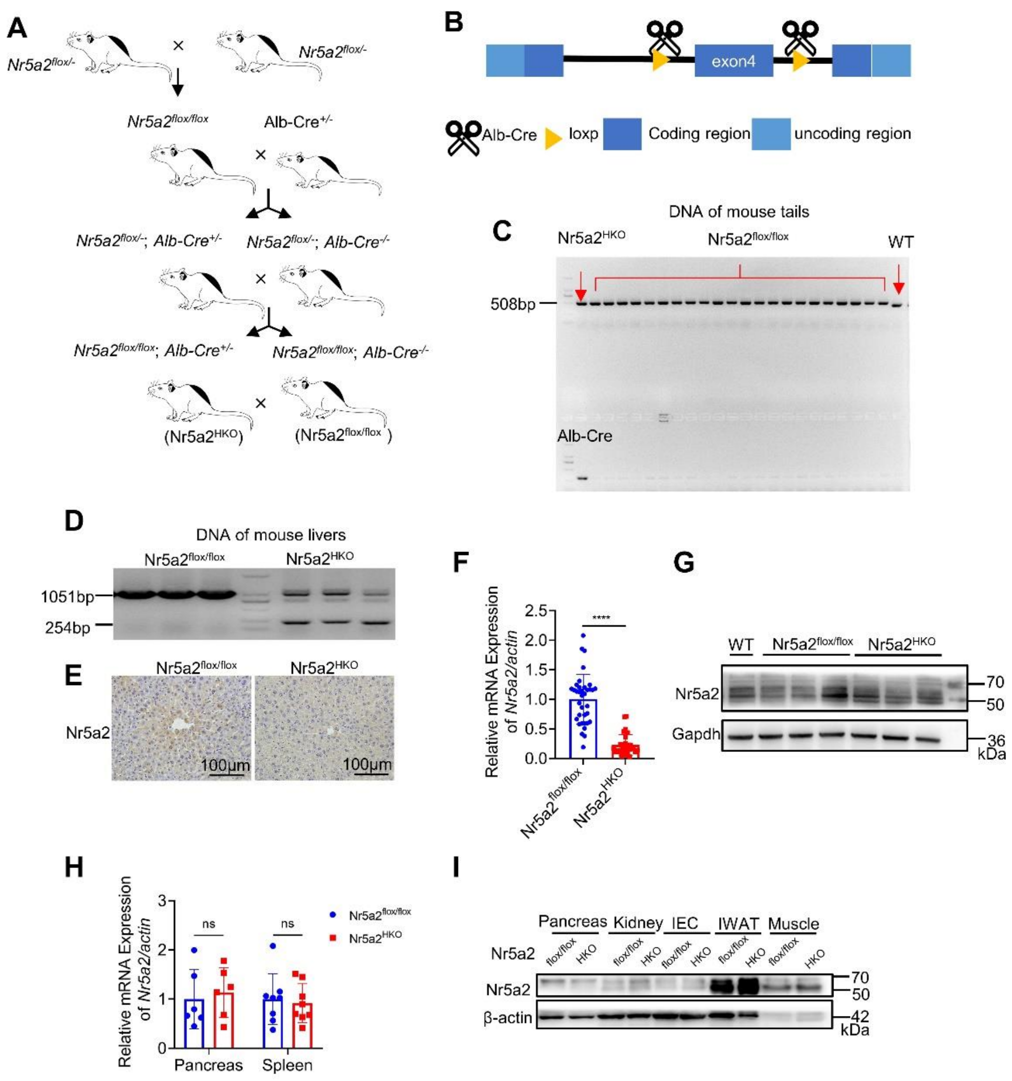

Figure S2. The hepatocyte-specific knockout mouse of NR5A2 was established.

(A) Breeding strategy for generating hepatocyte-specific knockout mice of Nr5a2 (Nr5a2^{HKO} mice) and their littermates.

(B) Schematic representation of Nr5a2 knockout in Nr5a2^{HKO} mice.

(C-D) Nr5a2^{HKO} mice were genotyped using tail DNA (C) and liver DNA (D). Tail:

$Nr5a2^{flox/flox}$ mice: 508bp, Alb-Cre: negative. Wild type mice:405bp, Alb-Cre: negative. $Nr5a2^{HKO}$ mice: 508bp, Alb-Cre: positive. Liver: $Nr5a2^{flox/flox}$ mice: 1051bp. $Nr5a2^{HKO}$ mice: 1051bp and 254bp (haploid Alb-Cre).

(E-G) Nr5a2 expression was detected by immunohistochemical staining (E), RT-qPCR (F) and western blotting (G) in the liver of $Nr5a2^{flox/flox}$ mice and $Nr5a2^{HKO}$ mice. N=36/group, ****$p < 0.0001$. Data are presented as means ± SD.

(H-I) Nr5a2 expression was detected in other organs via RT-qPCR (H) and western blotting (I) in the $Nr5a2^{flox/flox}$ mice and $Nr5a2^{HKO}$ mice (IEC: intestinal epithelial cell, IWAT: inguinal white adipose tissue). N=6-8/group, ns, not significant. Data are presented as means ± SD.

Figure S3

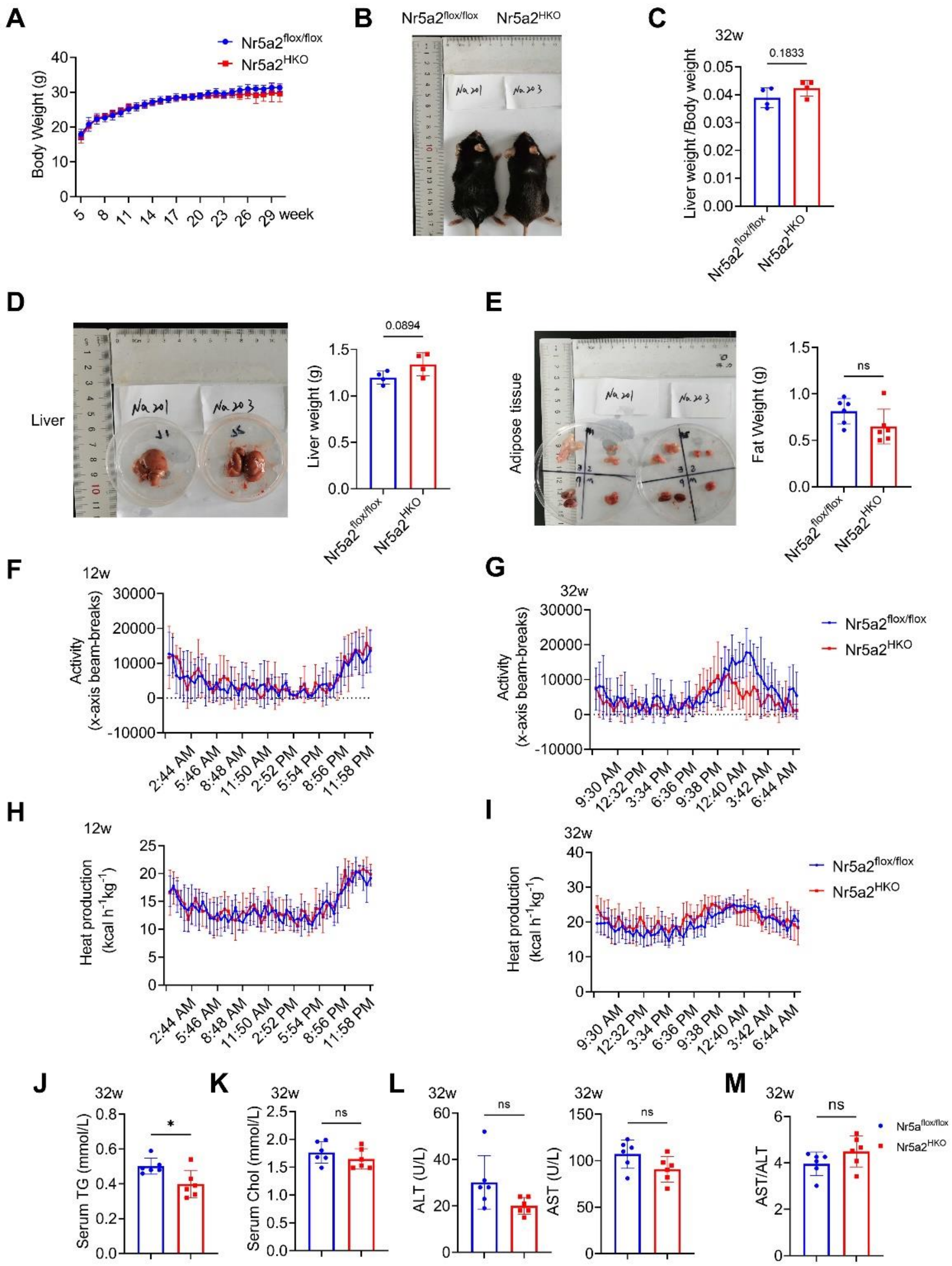
A
Nr5a2flox/flox
Nr5a2HKO
Body Weight (g)
week
B
Nr5a2flox/flox
Nr5a2HKO
C
32w
0.1833
Liver weight /Body weight
D
Liver
0.0894
Liver weight (g)
E
Adipose tissue
ns
Fat Weight (g)
F
12w
Activity (x-axis beam-breaks)
G
32w
H
12w
Heat production (kcal h-1kg-1)
I
32w
J
32w
Serum TG (mmol/L)
K
32w
ns
Serum Chol (mmol/L)
L
32w
ns
ALT (U/L)
AST (U/L)
M
32w
ns
AST/ALT
Nr5aflox/flox
Nr5a2HKO

Figure S3. The indifference phenotype in Nr5a2^{HKO} mice model.

Male Nr5a2$^{flox/flox}$ and Nr5a2^{HKO} mice were fed regular diet for 12 weeks and 32 weeks, N=6/group, data are presented as means ± SD.

(A) Body weight recording of 32-week-old Nr5a2^{HKO} mice model.

(B-E) Body shape under anesthesia (B), ratio of liver weight to body weight (C), appearance and weight of liver (D) and adipose tissue (E) in Nr5a2^{HKO} mice and their littermates at the age of 32 weeks (Na201: Nr5a2$^{flox/flox}$, Na203: Nr5a2^{HKO}). N=4/group (2 mice were excluded for liver perfusion with 4% polyformaldehyde dissolved in 0.01 M phosphate buffer solution), ns, not significant.

(F-G) The activity monitoring data in metabolism cage of 12-week-old (F) and 32-week-old (G) Nr5a2^{HKO} mice and their littermates.

(H-I) The heat production monitoring data in metabolism cage of 12-week-old (H) and 32-week-old (I) Nr5a2^{HKO} mice and their littermates.

(J-K) Serum TG (J) and cholesterol (K) of 32-week-old Nr5a2^{HKO} mice and their littermates. ns, not significant, *$p < 0.05$.

(L) ALT and AST of 32-week-old Nr5a2^{HKO} mice and their littermates. ns, not significant.

(M) The ratio of AST to ALT of 32-week-old Nr5a2^{HKO} mice and their littermates. ns, not significant.

Figure S4

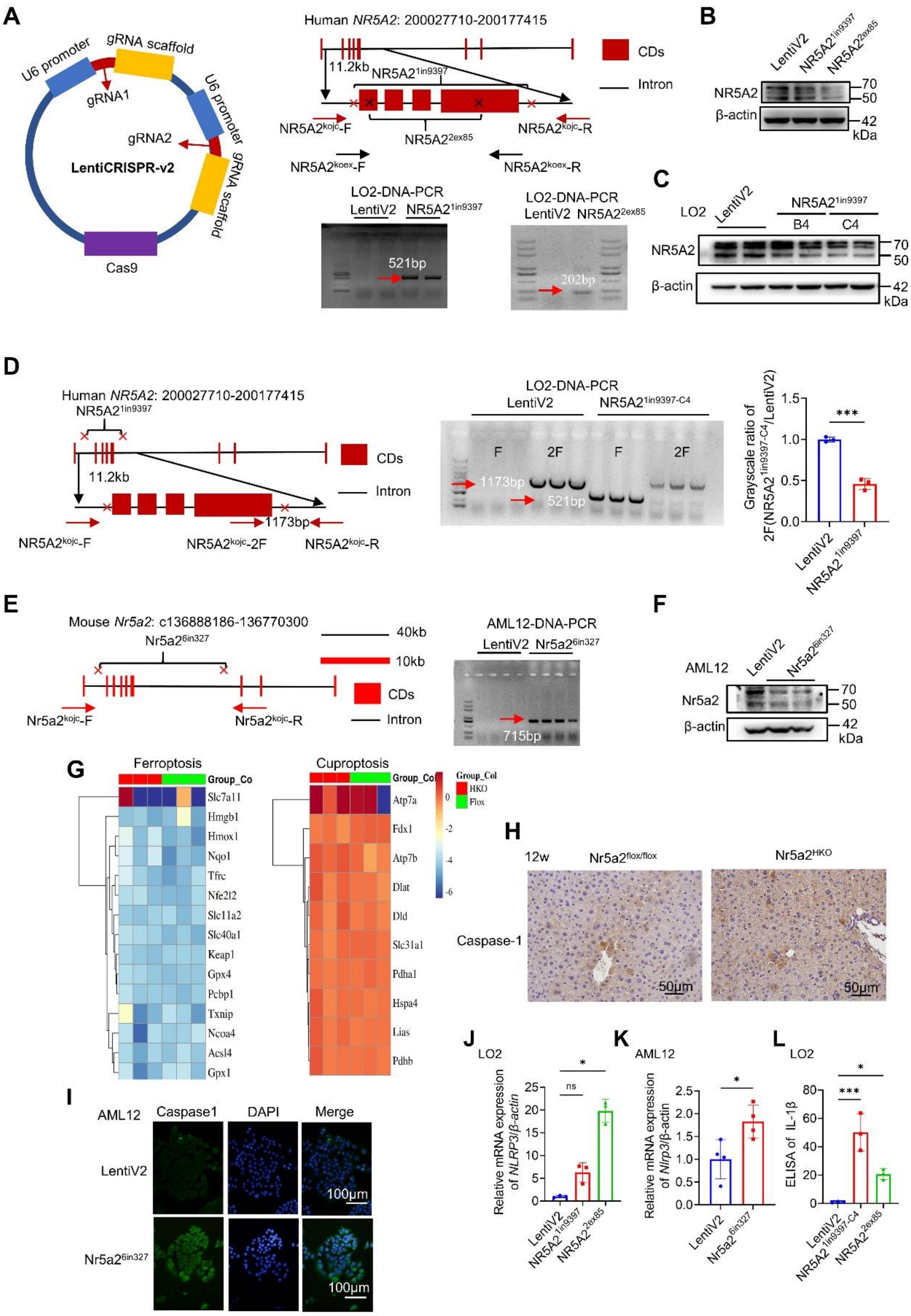
A
U6 promoter
gRNA scaffold
gRNA1
U6 promoter
gRNA2
LentiCRISPR-v2
gRNA scaffold
Cas9
Human NR5A2: 200027710-200177415
11.2kb
NR5A2 1in9397
CDs
Intron
NR5A2 kojc-F
NR5A2 2ex85
NR5A2 kojc-R
NR5A2 koex-F
NR5A2 koex-R
LO2-DNA-PCR
LentiV2 NR5A2 1in9397
521bp
LO2-DNA-PCR
LentiV2 NR5A2 2ex85
202bp
B
LentiV2
NR5A2 1in9397
NR5A2 2ex85
NR5A2
β-actin
70
50
42
kDa
C
LO2
LentiV2
NR5A2 1in9397
B4
C4
NR5A2
β-actin
D
Human NR5A2: 200027710-200177415
NR5A2 1in9397
11.2kb
CDs
Intron
1173bp
NR5A2 kojc-F
NR5A2 kojc-2F
NR5A2 kojc-R
LO2-DNA-PCR
LentiV2
NR5A2 1in9397-C4
F
2F
1173bp
521bp
Grayscale ratio of 2F(NR5A2 1in9397-C4/LentiV2)
***
LentiV2
NR5A2 1in9397
E
Mouse Nr5a2: c136888186-136770300
Nr5a2 6in327
40kb
10kb
CDs
Intron
Nr5a2 kojc-F
Nr5a2 kojc-R
AML12-DNA-PCR
LentiV2 Nr5a2 6in327
715bp
F
AML12
LentiV2
Nr5a2 6in327
Nr5a2
β-actin
G
Ferroptosis
Group_Co
Slc7a11
Hmgb1
Hmox1
Nqo1
Tfrc
Nfe2l2
Slc11a2
Slc40a1
Keap1
Gpx4
Pcbp1
Txnip
Ncoa4
Acsl4
Gpx1
Cuproptosis
Group_Co
Atp7a
Fdx1
Atp7b
Dlat
Dld
Slc31a1
Pdha1
Hspa4
Lias
Pdhb
Group_Col
HKO
Flox
H
12w
Nr5a2 flox/flox
Nr5a2 HKO
Caspase-1
50μm
I
AML12
Caspase1
DAPI
Merge
LentiV2
Nr5a2 6in327
100μm
J
LO2
Relative mRNA expression of NLRP3/β-actin
ns
*
LentiV2
NR5A2 1in9397
NR5A2 2ex85
K
AML12
Relative mRNA expression of Nlrp3/β-actin
*
LentiV2
Nr5a2 6in327
L
LO2
ELISA of IL-1β
***
*
LentiV2
NR5A2 1in9397-C4
NR5A2 2ex85

Figure S4. The deficiency of NR5A2 led to the induction of pyroptosis in liver cells.

(A) Identification of *NR5A2* knockout in the LO2 cell line genome. To achieve NR5A2 knockout in the LO2 cell line, two guide RNAs were designed to remove a long fragment of NR5A2. The guide RNAs, $NR5A2^{1in9397}$-gRNA1 and $NR5A2^{1in9397}$-gRNA2, inserted into LentiCRISPR-v2 and targeted NR5A2 intron 1 and intron 5 respectively. Primers located outside the cleavage site were utilized to confirm the successful removal of the long fragment ($NR5A2^{kojc}$-F/R). The presence of bands at 521bp indicates a successful knockout, whereas a failed knockout would result in an 11.2kb PCR product that may not be amplified. $NR5A2^{2ex85}$-gRNA1 and $NR5A2^{2ex85}$-gRNA2 targeted NR5A2 exon2 and exon 5, with verification design is similar to $NR5A2^{1in9397}$ ($NR5A2^{koex}$-F + $NR5A2^{koex}$-R:202bp).

(B) Western blotting analysis of NR5A2 in the LO2-$NR5A2^{1in9397}$ and LO2-$NR5A2^{2ex85}$ cell lines.

(C) Protein detection of NR5A2 in monoclonal cultivation of LO2-$NR5A2^{1in9397}$ cell line.

(D) Haploid knockout of NR5A2 in LO2-$NR5A2^{1in9397-C4}$ cell line was identified through DNA analysis. Different forward primers ($NR5A2^{kojc}$-F/2F) were designed and paired with the corresponding reverse primer ($NR5A2^{kojc}$-R). The total amount of DNA was consistent across all experimental groups ($NR5A2^{kojc}$-

2F + $NR5A2^{kojc}$-R:1173bp). N=3/group, ***$p < 0.001$. Data are presented as means ± SD.

(E) Design and identification of *Nr5a2* knockout in the AML12 cell line followed a similar approach as $NR5A2^{1in9397}$ ($Nr5a2^{kojc}$-F + $Nr5a2^{kojc}$-R: 715bp).

(F) Western blotting analysis of Nr5a2 in the AML12-$Nr5a2^{6in327}$ cell line.

(G) Heatmaps were generated to cluster genes involved in ferroptosis and cuproptosis pathways using RNA-seq data from 5-week-old $Nr5a2^{HKO}$ mice model, N=3/group.

(H) Immunohistochemical staining of Caspase1 expression patterns in the liver of 5-week-old $Nr5a2^{HKO}$ mice and their littermates.

(I) Immunofluorescence analysis demonstrated total Caspase1 levels in the AML12-$Nr5a2^{6in327}$ cell line compared to its negative control.

(J-K) *NLRP3* mRNA expression level was determined in the LO2-$NR5A2^{1in9397}$ (J), LO2-$NR5A2^{2ex85}$ (J) and AML12-$Nr5a2^{6in327}$ (K) cell lines, respectively. LO2: N=3/group, AML12: N=4/group, ns, not significant, *$p < 0.05$. Data are presented as means ± SD.

(L) ELISA of IL-1β in the cell supernatant of LO2-$NR5A2^{1in9397\text{-}C4}$, LO2-$NR5A2^{2ex85}$ and their control cell line LO2-LentiV2. N=3/group, *$p < 0.05$, ***$p < 0.001$. Data are presented as means ± SD.

Figure S5

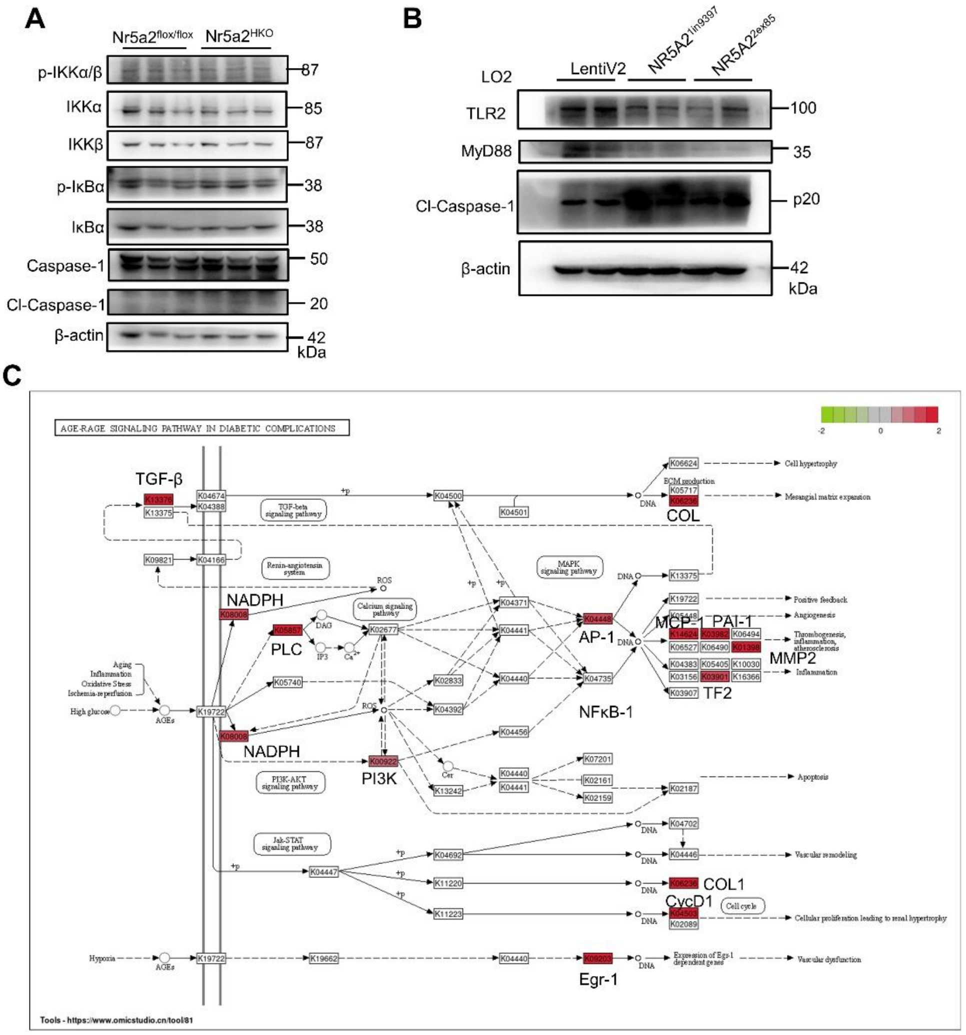

Figure S5. ROS serves as a prominent stimulus for the activation of the NF-κB pathway in hepatocytes with NR5A2 deficiency.

(A) Western blotting analysis was performed to examine the expression of molecules involved in the NF-κB pathway and pyroptosis in the livers of Nr5a2$^{flox/flox}$ and Nr5a2^{HKO} mice.

(B) The protein levels of TLR2 and MYD88, two key molecules involved in NF-

κB activation, were assessed in the LO2-NR5A2^{1in9397} and LO2-NR5A2^{2ex85} cell lines.

(C) OmicStudio tool was utilized to visualize the differential expression genes in the AGE-RAGE signaling pathway based on RNA-seq data from 5-week-old Nr5a2^{HKO} mice model (https://www.omicstudio.cn/tool).

Figure S6

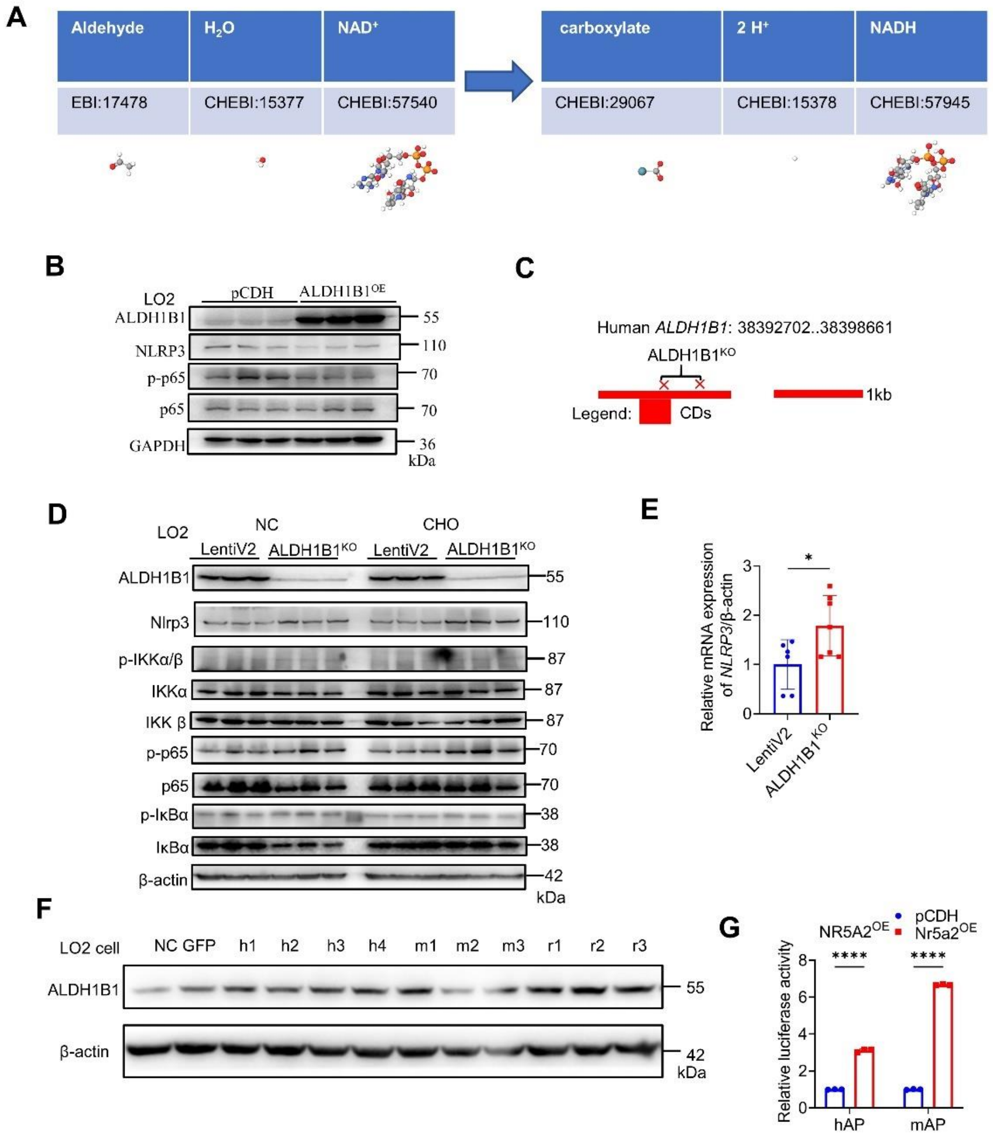

Figure S6. ALDH1B1 has the ability to inhibit NF-κB pathway and can be transcriptionally promoted by NR5A2.

(A) The conversion of aldehyde into carboxylate is facilitated by ALDH1B1.

(B) Western blotting was performed to detect the activation of the NF-κB pathway in the LO2 cell line overexpressing ALDH1B1 (pCDH refers to pCDH_EF1a-IRES-puro_BsrG1 vector).

(C) A schematic diagram illustrating the design of *ALDH1B1* knockout in the LO2 cell line (LO2-ALDH1B1^{KO}).

(D) Western blotting was performed to detect the activation of the of the NF-κB pathway in the LO2-ALDH1B1^{KO} cell line.

(E) The mRNA level of *NLRP3* was assessed in the LO2-ALDH1B1^{KO} cell line. N=6-7/group, *p < 0.05. Data are presented as means ± SD.

(F) The ALDH1B1 was upregulated in LO2 cell line overexpressing NR5A2. The samples are the same as those used in figures S1D and S1E.

(G) The transcriptional activity of NR5A2 on the promoter region *of ALDH1B1* was analyzed using a dual-luciferase reporter system. NR5A2^{OE} refers to overexpression of human and mouse full-length *NR5A2* in the HEK293T cell line, respectively. h(m)AP: human (mouse) *ALDH1B1* promoter region. N=3/group, ****p < 0.0001. Data are presented as means ± SD.

Table S1 Basic information of included patients

Item	Control	NASH
Sample size	6	6
Age (years)	32.33 (3.921)	31.67 (3.630)
Body mass index (kg/m2)	29.08 (2.193)	38.33 (3.446)
AST (U/L)	54.25 (28.26)	59.13 (16.17)
ALT (U/L)	27.5 (5.309)	114.3 (28.49)
Triglycerides (mmol/L)	1.278 (0.1549)	4.287 (1.264)
Total cholesterol (mmol/L)	4.698 (0.3887)	5.477 (0.5503)
HDL-C (mmol/L)	1.092 (0.141)	0.805 (0.0746)
LDL-C (mmol/L)	2.663 (0.3006)	2.94 (0.2817)

[a.] The inclusion criteria of NASH patients were as follows: a) The recommended alcohol intake is less than 20g per week for men and less than 10g per week for women. b) liver biopsy shows that there is steatosis with or without fibrosis. c) hepatitis virus is negative.

Table S2 Primer sequences for PCR

Primer name	Primer sequence （5’ to 3’）
Nr5a2_P1	TTTATACTTTGCAACGCTTCTCC
Nr5a2_P2	AATGTCGCCTTTGCCTAATGA
Nr5a2_P5	GTGCCAGTCCCTTACAGC
Nr5a2_P6	GTTCCAACTTGCAGCACAGATTC
Mus-Cre-F	CAGCATTGCTGTCACTTGGTC
Mus-Cre-R	ATTTGCCTGCATTACCGGTCG
NR5A2^{kojc}-F	CATGGACCAAGAAGTCGCTA
NR5A2^{kojc}-2F	CTGTAAGGGCCGACCGAATGCG
NR5A2^{kojc}-R	CCAACCTAATGTCAAGGCAA
NR5A2^{koex}-F	GCTGGGCTTCCGGACCGAC
NR5A2^{koex}-R	CTTAAGTTCTCTGAAGAAG
Nr5a2^{kojc}-F	ATGATTTCTGCTGTAAGCCAAAG
Nr5a2^{kojc}-R	GTTCTCCAGTAACCAGGAAGATTG
CDS-hALDH1B1-F:	ATGCTGCGCTTCCTGGCACC
CDS-hALDH1B1-R:	TTACGAGTTCTTCTGAGGAACC

Table S3 gRNA sequences for gene knockout

gRNA	gRNA sequence （5’ to 3’）
NR5A2^{1in9397}-gRNA1	GCCTCTGCTTGGTGAAGCCGG
NR5A2^{1in9397}- gRNA2	GTGTAGCTCTTAGTTTGGGG
NR5A2^{2ex85}- gRNA1	GGGACTGGCTCGATCGCATG
NR5A2^{2ex85}- gRNA2	GGCCTATTTGCAGCAAGAGC
Nr5a2^{6in327}- gRNA1	GGGTCTGTTAAGGACCATTC
Nr5a2^{6in327}- gRNA2	GCTAAGTGGATCTAATACAA
ALDH1B1^{KO}- gRNA1	GGTAGGGTTGACCGTCGGGA
ALDH1B1^{KO}- gRNA2	GAAGCCCTGTTCTTCAACAT

Table S4 Primer sequences for clone

Primer name	Primer sequence （5’ to 3’）
pCDH-Flag	GCTGTGACCGGCGCCTACTCTAGACAACATGGATTACAAGGACGACGATGACAAG
Flag-hNR5A2-Iso1-F	ACAAGGACGACGATGACAAGTCTTCTAATTCAGATACTGG
hNR5A2- Iso2-R	CCATTTTAAATTGAGACACAATAGGTGTAAGTCCGTG
hNR5A2- Iso2-F	CACGGACTTACACCTATTGTGTCTCAATTTAAAATGG
Flag-hrNR5A2-Iso3/X2-F	ACAAGGACGACGATGACAAGGTGAATTACTCCTATGATGAAGATCTG
Flag-hmNR5A2-IsoX3/2-F	ACAAGGACGACGATGACAAGCTGCCCAAAGTGGAGACGG
pCDH-hNR5A2-R	GTACACTCGAGAGCGCTGGATCCTCATGCTCTTTTGGCATGCAACATTTC
Flag-mNr5a2-Iso1-F	ACAAGGACGACGATGACAAG TCTGCTAGTTTGGATACTG
mNR5A2- IsoX1-R	GGTCCGGAAGCCCAGCACCAATAGCTGTAAGTC
mNR5A2- IsoX1-F	GACTTACAGCTATTGGTGCTGGGCTTCCGGACC
pCDH-mNr5a2-R	GTACACTCGAGAGCGCTGGATCCTCAGGCTCTTTTGGCATGCAGC
Flag-rNr5a2-F	ACAAGGACGACGATGACAAGTCTGCTAGTTCGATTACTGG
pCDH-rNr5a2-R	GTACACTCGAGAGCGCTGGATCCTCAGGCTCTTTTGGCGTGCAGC
PCDH-Flag-hALD-F	ACAAGGACGACGATGACAAGCTGCGCTTCCTGGCAC

PCDH-hALD-R	GTACACTCGAGAGCGCTGGATCCTCACG AGTTCTTCTGAGGAAC
PCDH-Flag-mAld-F	ACAAGGACGACGATGACAAGCTGACTGC CCGACTCTTGCTG
PCDH-mAld-R	GTACACTCGAGAGCGCTGGATCCTCAGG AATTCTTCTCGGGAAC
pCDH-neo-Flag-F	GAATTCGAATTTAAATCGGATCCGCCACC ATGGATTACAAGGACGACGATGACAAG
pCDH-neo-hNR5A2-R	GGGAGGGAGAGGGGCGCGGCCGCTGTA CATCATGCTCTTTTGGCATGCAACATTTC
pCDH-neo-mNr5a2-R	GGGAGGGAGAGGGGCGCGGCCGCTGT ACATCAGGCTCTTTTGGCATGCAGC
pCDH-neo-GFP- F	GAATTCGAATTTAAATCGGATCCGCCACC ATGGTGAGCAAGGGCGAGGAG
pCDH-neo-GFP-R	GGGAGGGAGAGGGGCGCGGCCGCTGTA CATCACTTGTACAGCTCGTCCATG
pCDH-neo-hALD-R	GGGAGGGAGAGGGGCGCGGCCGCTGTA CATCACGAGTTCTTCTGAGGAAC
pCDH-neo-mAld-R	GGGAGGGAGAGGGGCGCGGCCGCTGTA CATCAGGAATTCTTCTCGGGAAC
mAP-F	CTCTGGCCTAACTGGCCGGTACCCAACCA AGAACTCTGCAGG
mAP-R	CAAGCTTACTTAGATCGCAGATCTCTAGTAT CAGCGTTCTCCGGTCAG
2mAP-F	CTCTGGCCTAACTGGCCGGTACCCCTGAC AGCTTCAGTTACAGGGACAG
2mAP-R	CTGTCCCTGTAACTGAAGCTGTCAGGGGT ACCGGCCAGTTAGGCCAGAG
3mAP-F	CTCTGGCCTAACTGGCCGGTACCCCAGCA GTAGGGGATTGCACAATAGC
3mAP-R	GCTATTGTGCAATCCCCTACTGCTGGGGTA CCGGCCAGTTAGGCCAGAG
mAP-mut1-F	GTCCATGGCGTAGAGCGGAGC
mAP-mut1-R	GCTCCGCTCTACGCCATGGAC
mAP-mut3-F	GGTGTCGTACAAATCCAC
mAP-mut3-R	GTGGATTTGTACGACACC
hAP-F	CTCTGGCCTAACTGGCCGGTACCATTCCTC TGTATTCTTCACTGGTC
hAP-R	CAAGCTTACTTAGATCGCAGATCTGCAGGC TCGGGCTCCGGTTC
2hAP-F	CTCTGGCCTAACTGGCCGGTACCGCGTGC CCGCCTTCCACATCCGG
2hAP-R	CCGGATGTGGAAGGCGGGCACGCGGTAC CGGCCAGTTAGGCCAGAG

3hAP-F	CTCTGGCCTAACTGGCCGGTACCCGGCGA CAGGACGTAGGCAGC
3hAP-R	GCTGCCTACGTCCTGTCGCCGGGTACCGG CCAGTTAGGCCAGAG
hAP-mut2-F	GGGAGGAGCGGCGTAGGCCGGCGACAG
hAP-mut2-R	CTGTCGCCGGCCTACGCCGCTCCTCCC
hAP-mut3-F	GACAGGACGTACTCAGCGCCCCAG
hAP-mut3-R	CTGGGGCGCTGAGTACGTCCTGTC
hAP-mut3-1F	GGCAGCGCCCCACTCGCAGGCGGAG
hAP-mut3-1R	CTCCGCCTGCGAGTGGGGCGCTGCC

Table S5 Antibodies and materials

ANTIBODIES	SOURCE	IDENTIFIER
Anti-NR5A2, mouse monoclonal	R&D systems	Cat# PPH2325
Anti-NR5A2, goat polyclonal	Everest	Cat# EB12283
Anti-ALDH1B1, mouse monoclonal	Santa Cruz	Cat# SC-393583
Anti-NLRP3, rabbit polyclonal	Proteintech	Cat# 19771-1-AP
Anti-Phospho-NF-κB p65 (Ser536), rabbit recombinant monoclonal, 93H1	Cell Signaling Technology	Cat# 3033
Anti-NF-κB p65, rabbit monoclonal, D14E12	Cell Signaling Technology	Cat# 8242
Anti-Caspase-1, rabbit polyclonal	Cell Signaling Technology	Cat# 2225
Anti-Caspase-1, rabbit polyclonal	Proteintech	Cat# 22915-1-AP
Anti-Phospho-IKKα/β(Ser176/180), rabbit monoclonal, 16A6	Cell Signaling Technology	Cat# 2697
Anti-IKKα, rabbit monoclonal	Cell Signaling Technology	Cat# 2682
Anti-IKKβ, rabbit monoclonal, 2C8	Cell Signaling Technology	Cat# 2370
Anti-Phospho-IκBα (Ser32), rabbit monoclonal, 14D4	Cell Signaling Technology	Cat# 2859
Anti-IκBα, mouse monoclonal, L35A5	Cell Signaling Technology	Cat# 4814
Anti-GSDMD, rabbit monoclonal	Abcam	Cat# ab209845
Anti-Pro IL-1β, rabbit polyclonal	Proteintech	Cat# 16806-1-AP
Anti-mature IL-1β, rabbit monoclonal	Abways	Cat# CY5087
Cleaved-IL-1β, rabbit polyclonal	Affinity	Cat #AF4006
Anti-GAPDH, rabbit monoclonal	BOSRER	Cat# BM3874

Anti-β-actin, mouse monoclonal	ZENBIO	Cat# 200068-8F10
Anti-MyD88, rabbit polyclonal	Immunoway	Cat# YT2928
Anti-TLR2, rabbit monoclonal	Abcam	Cat# Ab191458
CoraLite488-conjugated Goat Anti-Mouse IgG(H+L), polyclonal	Proteintech	Cat# SA00013-1
CoraLite594 – conjugated Goat Anti-Rabbit IgG(H+L), polyclonal	Proteintech	Cat# SA00013-4

MATERIALS	SOURCE	IDENTIFIER
Bacterial and Virus Strains		
DH5α Competent Cells	sangon	B528413

Experimental Models: Cell Lines		
LO2	ATCC, Gift from Yilei Zhang	Cat# CRL-2706
AML12	ATCC	Cat# CRL-2254
293T	ATCC	Cat# CRL-11268

Recombinant DNA		
pCDH_EF1a-IRES-puro_BsrG1	Gift from Lianying Jiao	N/A
pCDH-EF1a-Flag-hNR5A2-Iso1-puro	This Study	N/A
pCDH-EF1a-Flag-hNR5A2-Iso2-puro	This Study	N/A
pCDH-EF1a-Flag-hNR5A2-Iso3/X4-puro	This Study	N/A
pCDH-EF1a-Flag-hNR5A2-IsoX3-puro	This Study	N/A
pCDH-EF1a-Flag-mNr5a2-Iso1-puro	This Study	N/A
pCDH-EF1a-Flag-mNr5a2-Iso2/X2-puro	This Study	N/A
pCDH-EF1a-Flag-mNr5a2-IsoX1-puro	This Study	N/A
pCDH-EF1a-Flag-rNr5a2-Iso1-puro	This Study	N/A
pCDH-EF1a-Flag-rNr5a2-IsoX1-puro	This Study	N/A
pCDH-EF1a-Flag-rNr5a2-IsoX2-puro	This Study	N/A
pCDH-EF1a-Flag-hALD-puro	This Study	N/A
pCDH-EF1a-Flag-mAld-puro	This Study	N/A
pCDH-CMV-MCS-EF1-Neo	Gift from Lianying Jiao	N/A
pCDH-CMV-Flag-hNR5A2-Iso1-Neo	This Study	N/A
pCDH-CMV-Flag-hNR5A2-Iso2-Neo	This Study	N/A
pCDH-CMV-Flag-hNR5A2-Iso3/X4-Neo	This Study	N/A
pCDH-CMV-Flag-hNR5A2-IsoX3-Neo	This Study	N/A
pCDH-CMV-Flag-mNr5a2-Iso1-Neo	This Study	N/A

pCDH-CMV-Flag-mNr5a2-Iso2/X2-Neo	This Study	N/A
pCDH-CMV-Flag-mNr5a2-IsoX1-Neo	This Study	N/A
pCDH-CMV-GFP-Neo	Gift from Lianying Jiao	N/A
pCDH-CMV-Flag-hALD-Neo	This Study	N/A
pCDH-CMV-Flag-mAld-Neo	This Study	N/A
lentiCRISPR v2	Addgene, Gift from Lianying Jiao	RRID:Addgene_52961
pGL4.35[*luc2P*/9X*GAL4*UAS/Hygro] Vector	Promega	Cat# E1370
pGL4.35-hAP	This Study	N/A
pGL4.35-2hAP	This Study	N/A
pGL4.35-3hAP	This Study	N/A
pGL4.35-mAP	This Study	N/A
pGL4.35-2mAP	This Study	N/A
pGL4.35-3mAP	This Study	N/A
pGL4.35-hAP-mut2	This Study	N/A
pGL4.35-hAP-mut3	This Study	N/A
pGL4.35-mAP-mut1	This Study	N/A
pGL4.35-mAP-mut3	This Study	N/A
pRL-TK vector	Addgene	Cat# E2241
psPAX2	Addgene, Gift from Lianying Jiao	RRID:Addgene_12260
pMD2.G	Addgene, Gift from Lianying Jiao	RRID:Addgene_12259

Table S6 Primer sequences for RT-qPCR

Primer name	Primer sequence （5’ to 3’）
Mus_Nr5a2-P1	TGTGGCGATAAAGTGTCTGGG
Mus_Nr5a2-P2	ATCGACAGTAGGGACATCGT
Homo_NR5A2-F	CTTTGTCCCGTGTGTGGAGAT
Homo_NR5A2-R	GTCGGCCCTTACAGCTTCTA
Mus_Nr5a2-F	GGGGCAGAAATAAGTTTGGGC
Mus_Nr5a2-R	TTGGAGGCGGAATGAATGTTC
Aldh1b1-mus-F	GACATGGAGCATGCCGTAGA
Aldh1b1-mus-R	GAGAAGCTTTGCCCCTTCCT
ALDH1B1-homo-F	GGTGAACATCATCACGGGGT
ALDH1B1-homo-R	GAATCGCCAGCTGCTTTCTG
SLC27A1-homo-F	ATGGCTATGTCAGCGAGAGC
SLC27A1-homo-R	CTGGAACAGCCACCCCATAG

hum-actin-F	GAGAAAATCTGGCACCACACC
hum-actin-R	GGATAGCACAGCCTGGATAGCAA
Mus-actin-F	GGCTGTATTCCCCTCCATCG
Mus-actin-R	CCAGTTGGTAACAATGCCATGT
hum-TGFβ-F	GGCCAGATCCTGTCCAAGC
hum-TGFβ-R	GTGGGTTTCCACCATTAGCAC
Slc27a1-mus-F	CGCTTTCTGCGTATCGTCTG
Slc27a1-mus-R	GATGCACGGGATCGTGTCT
Got2-mus-F	GGACCTCCAGATCCCATCCT
Got2-mus-R	GGTTTTCCGTTATCATCCCGGTA
CD36-mus-F	GGAGCCATCTTTGAGCCTTCA
CD36-mus-R	GAACCAAACTGAGGAATGGATCT
Fasn-mus-F	GGAGGTGGTGATAGCCGGTAT
Fasn-mus-R	TGGGTAATCCATAGAGCCCAG
Scd1-mus-F	TTCTTGCGATACACTCTGGTGC
Scd1-mus-R	CGGGATTGAATGTTCTTGTCGT
Dgat 2-mus-F	GCGCTACTTCCGAGACTACTT
Dgat2 -mus-R	GGGCCTTATGCCAGGAAACT
Ppara-mus-F	AGAGCCCCATCTGTCCTCTC
Ppara-mus-R	ACTGGTAGTCTGCAAAACCAAA
Cpt1a-mus-F	CTCCGCCTGAGCCATGAAG
Cpt1a-mus-R	CACCAGTGATGATGCCATTCT
Acadl-mus-F	TCTTTTCCTCGGAGCATGACA
Acadl-mus-R	GACCTCTCTACTCACTTCTCCAG
Acadm-mus-F	AGGGTTTAGTTTTGAGTTGACGG
Acadm-mus-R	CCCCGCTTTTGTCATATTCCG
Ucp2-mus-F	ATGGTTGGTTTCAAGGCCACA
Ucp2-mus-R	CGGTATCCAGAGGGAAAGTGAT
Acox1-mus-F	GGATGGTAGTCCGGAGAACA
Acox1-mus-R	AGTCTGGATCGTTCAGAATCAAG
Srebf1-mus-F	GCAGCCACCATCTAGCCTG
Srebf1-mus-R	CAGCAGTGAGTCTGCCTTGAT
Hmgcr-mus-F	AGCTTGCCCGAATTGTATGTG
Hmgcr-mus-R	TCTGTTGTGAACCATGTGACTTC
Cyp7a1-mus-F	GGGATTGCTGTGGTAGTGAGC
Cyp7a1-mus-R	GGTATGGAATCAACCCGTTGTC
Abcg5-mus-F	AGGGCCTCACATCAACAGAG
Abcg5-mus-R	GCTGACGCTGTAGGACACAT
Tnfα-mus-F	ATCGGTCCCCAAAGGGAT
Tnfα-mus-R	TGTCTTTGAGATCCATGC
Il1β-mus-F	CAGGCAGTATCACTCATT
Il1β-mus-R	GGAGCCTGTAGTGCAGTT
Tgfβ-mus-F	AACTATTGCTTCAGCTCC
Tgfβ-mus-R	CAGAAGTTGGCATGGTAGC

α-Sma-mus-F	CCAACCGGGAGAAAATGA
α-Sma-mus-R	CCAGACGCATGATGGCAT
Mmp2-mus-F	CCCTTCACTTTCCTGGGCAA
Mmp2-mus-R	AAGTTCTTGGTGTAGGTGTA
Timp2-mus-F	TGAACCACAGGTACCAGA
Timp2-mus-R	TCTTGATGCAGGCGAAGAA
Ccl2-mus-F	GATCCCAATGAGTAGGCT
Ccl2-mus-R	GATGCATTAGCTTCAGAT
Nlrp3-mus-F	ATTACCCGCCCGAGAAAGG
Nlrp3-mus-F	TCGCAGCAAAGATCCACACAG
NLRP3-homo-F	CGTGAGTCCCATTAAGATGGAGT
NLRP3-homo-R	CCCGACAGTGGATATAGAACAGA
