# Supplementary material for: Hepatocyte-specific NR5A2 deficiency induces pyroptosis and exacerbates non-alcoholic steatohepatitis by downregulating ALDH1B1 expression
Source: Cell Death Dis. 2024 Oct 23;15(10):770. doi: 10.1038/s41419-024-07151-1 (PMC11496806; doi:10.1038/s41419-024-07151-1)
Supplement: Supplementary file 2 — Original data [file 41419_2024_7151_MOESM2_ESM.docx]

**Supplemental Information**

**Hepatocyte-specific NR5A2 Deficiency Induces Pyroptosis and Exacerbates Non-alcoholic Steatohepatitis by Downregulating ALDH1B1 Expression**

Rong Zhao, Zizhen Guo, Kaikai Lu, Qian Chen, Farooq Riaz, Yimeng Zhou, Luyun Yang, Xiaona Cheng, Litao Wu, Kexin Cheng, Lina Feng, Sitong Liu, Xiaodan Wu, Minghua Zheng, Chunyan Yin, Dongmin Li

**Original western blots of figures**

Figure 1 A and 1 D


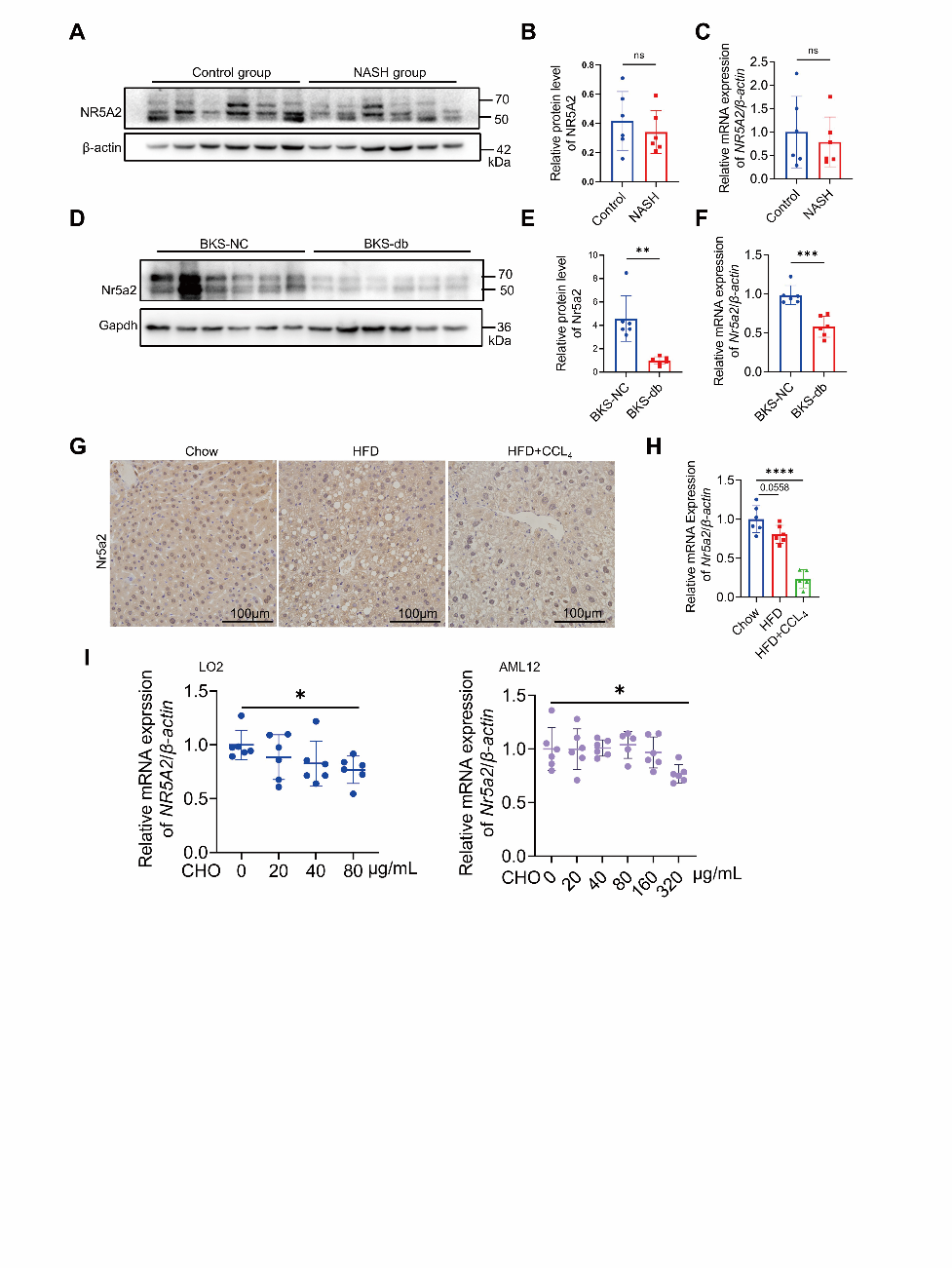

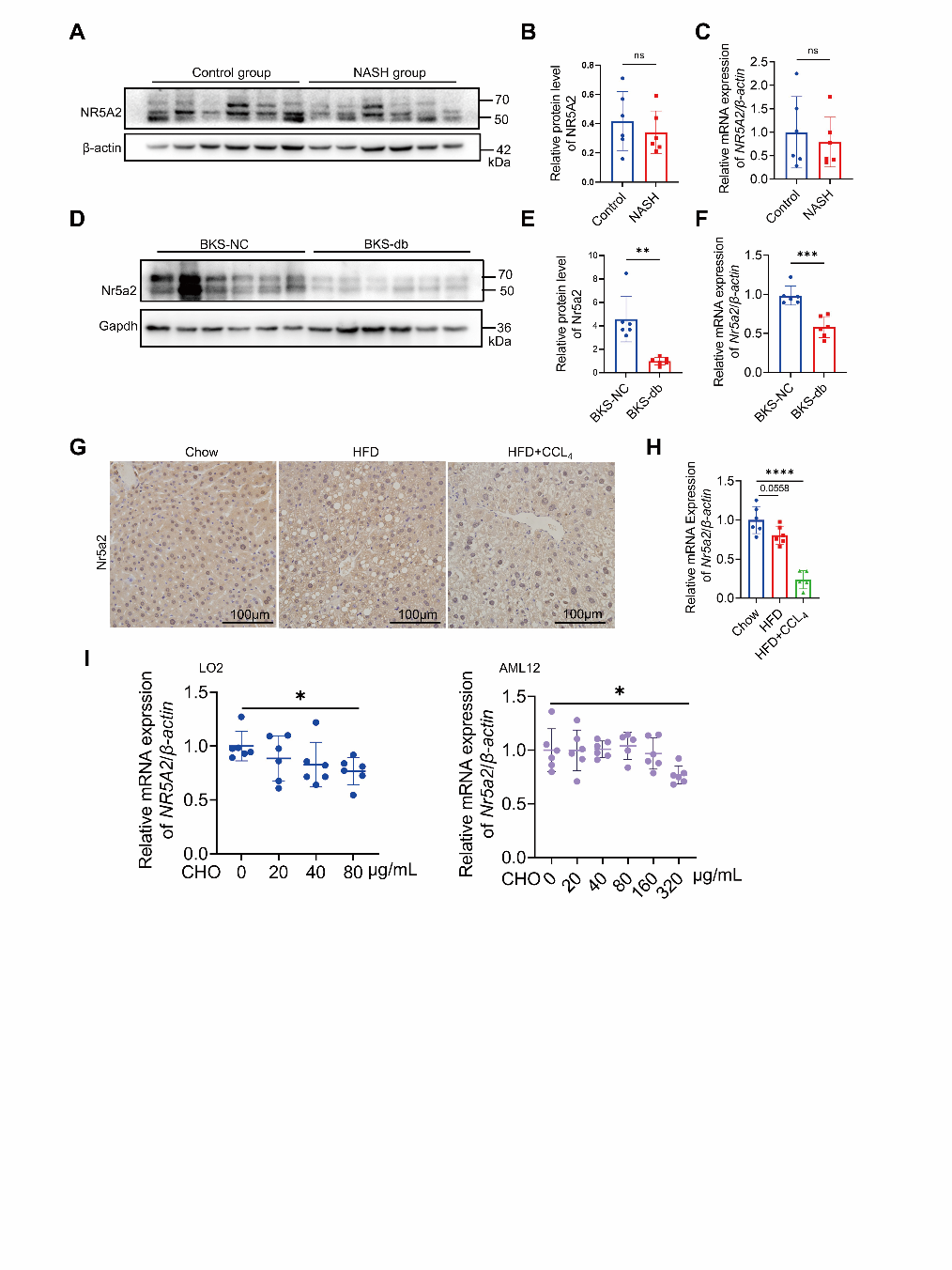


| 1A | NR5A2 | 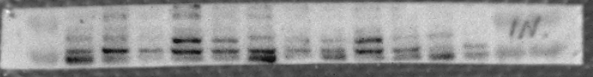 |
| --- | --- | --- |
|  | β-actin | 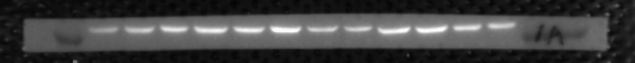 |
| 1D | Nr5a2 | 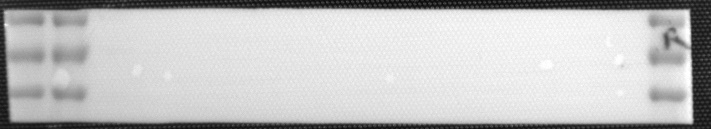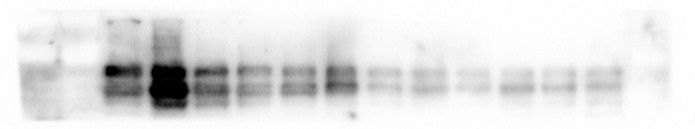 |
|  | Gapdh | 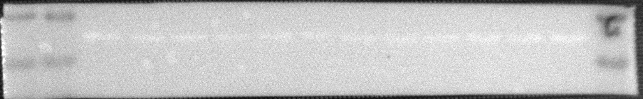 |

Figure 3F, 3J and 3K


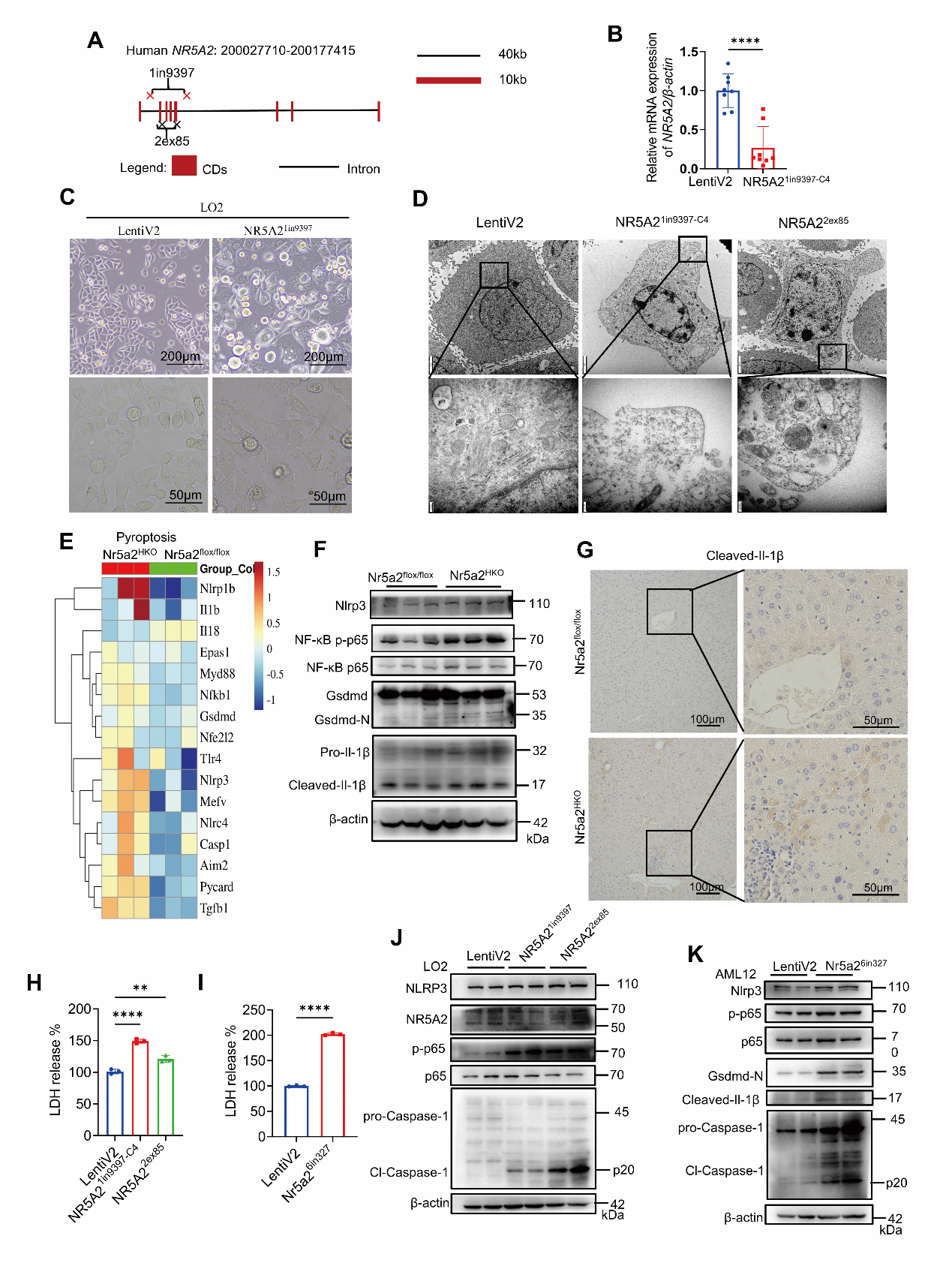

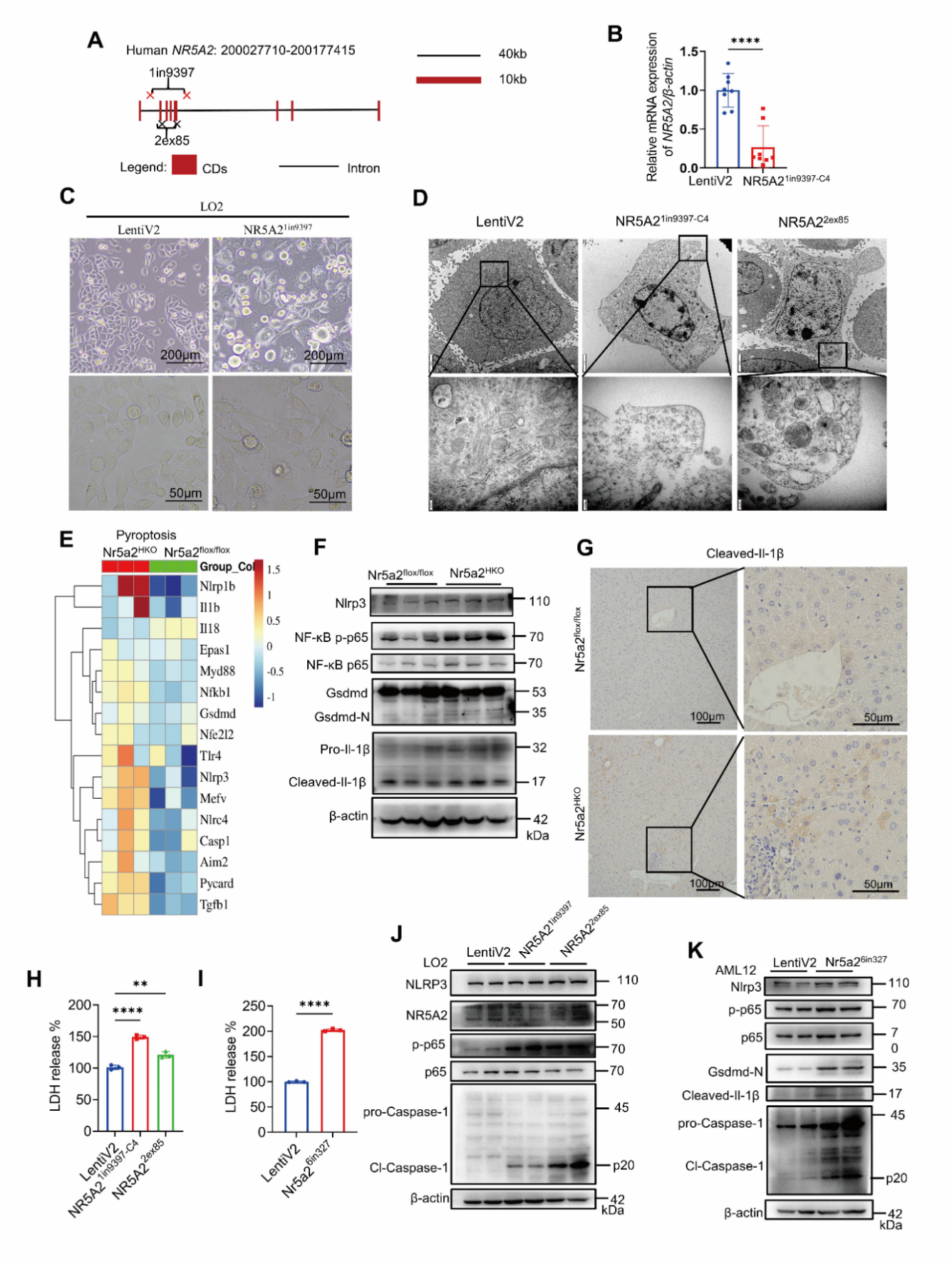

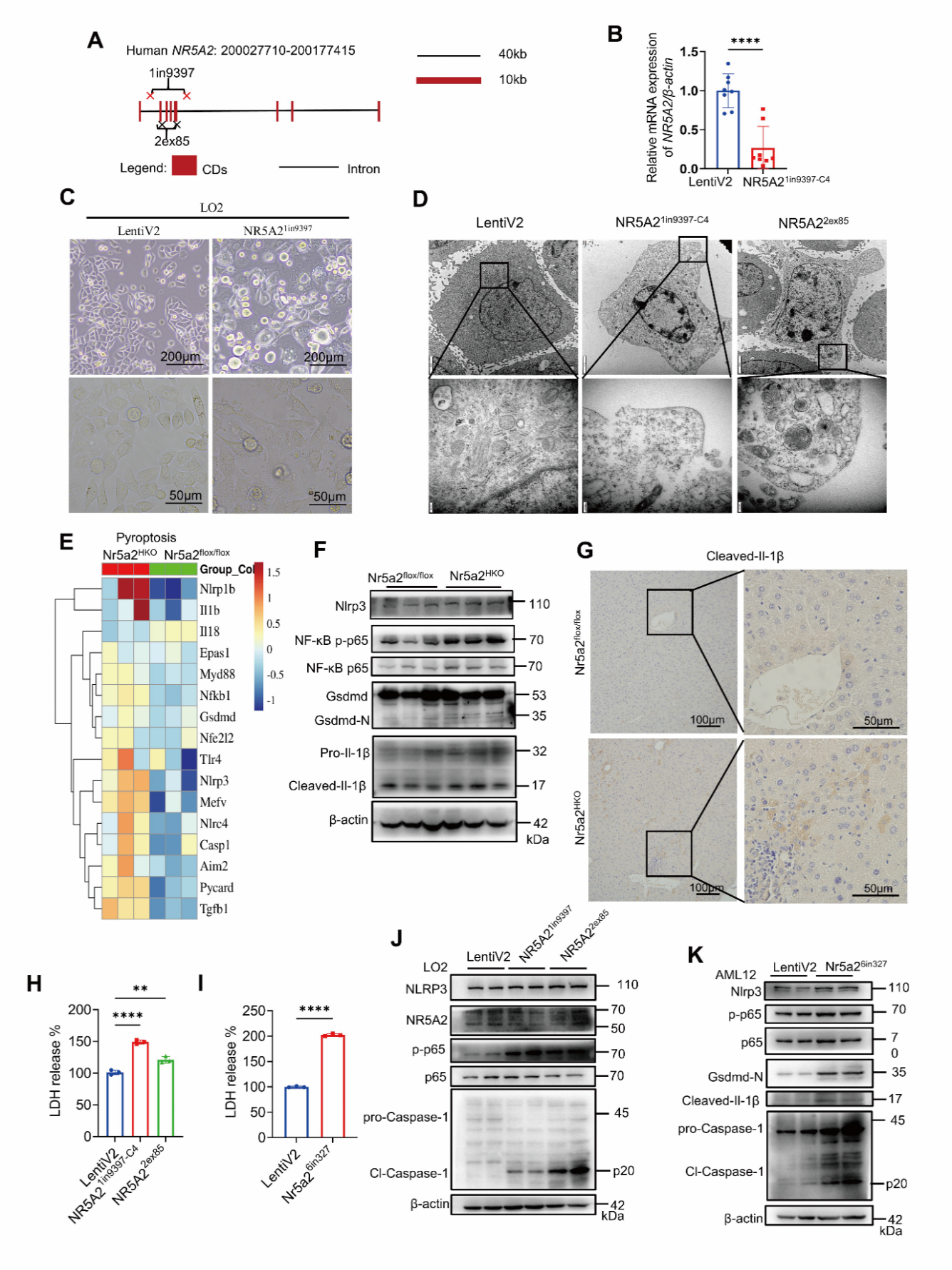


| 3F | | 3J | | 3K | |
| --- | --- | --- | --- | --- | --- |
| Nlrp3 | 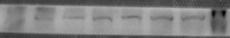 | NLRP3 | 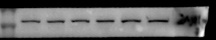 | Nlrp3 | 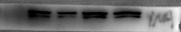 |
| NF-κB p-p65 | 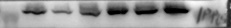 | NF-κB p-p65 | 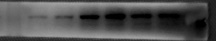 | NF-κB p-p65 | 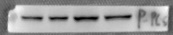 |
| NF-κB p65 | 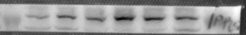 | NF-κB p65 | 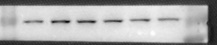 | NF-κB p65 | 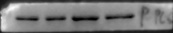 |
| β-actin | 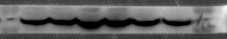 | β-actin | 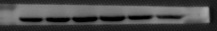 | β-actin | 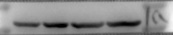 |
| Gsdmd | 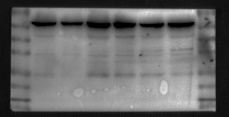 |  |  | Gsdmd | 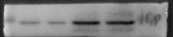 |
|  |  | Caspase-1 | 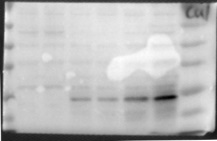 | Caspase-1 | 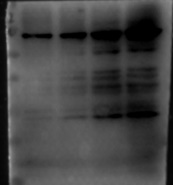 |
| Pro-Il-1β  Cleaved Il-1β | 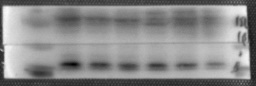 | NR5A2 | 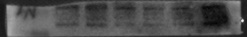 | Cleaved Il-1β | 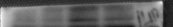 |

The pro-Il-1β and cleaved Il-1β were detected with different antibodies, so the same PVDF membrane was cut apart.

Figure 4A and 4B


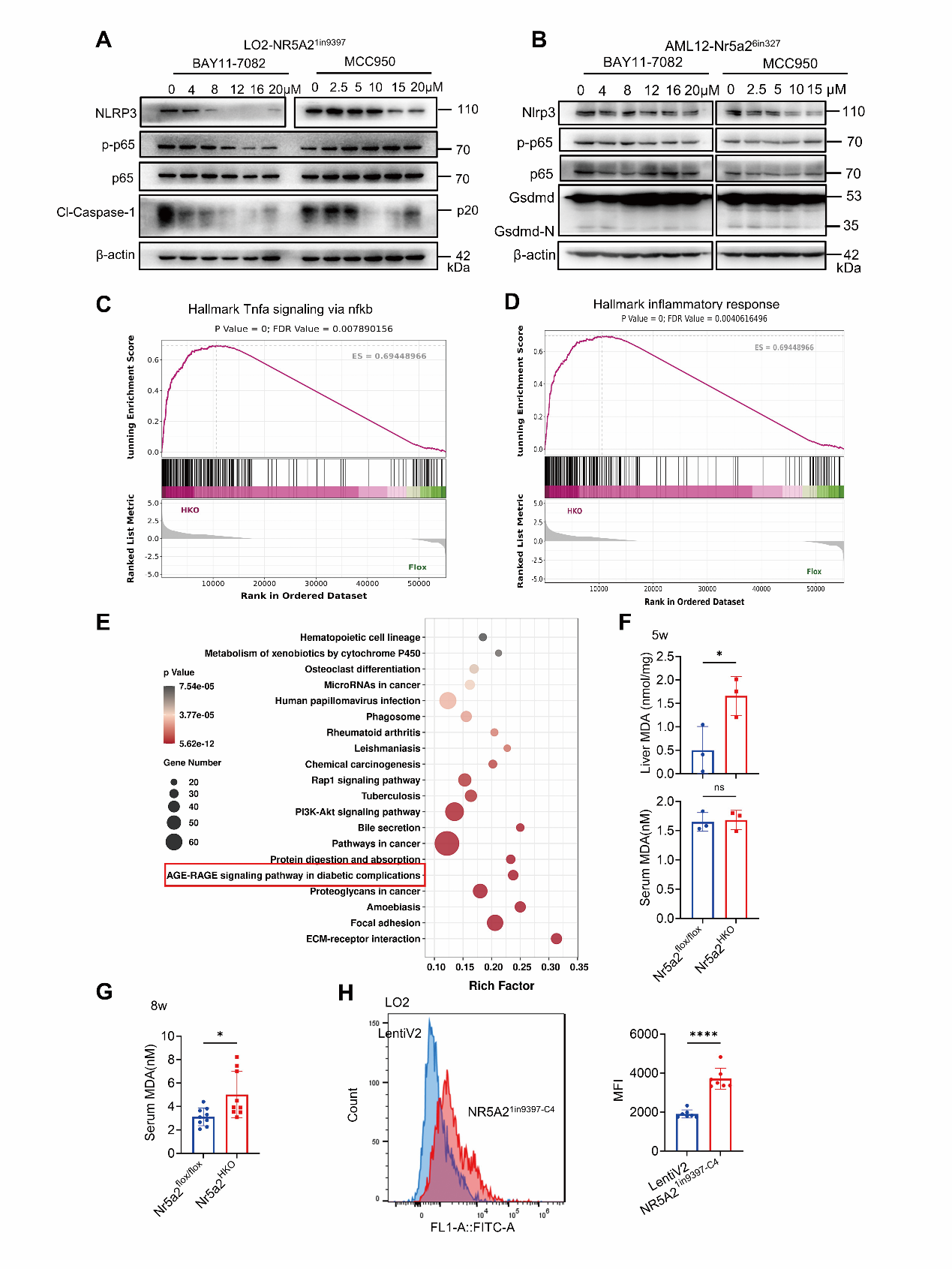


| 4A | | 4B | |
| --- | --- | --- | --- |
| NLRP3 | 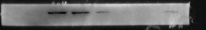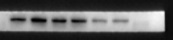 | Nlrp3 | 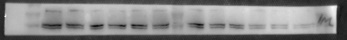 |
| NF-κB p-p65 | 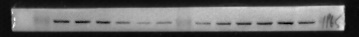 | NF-κB p-p65 | 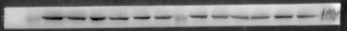 |
| NF-κB p65 | 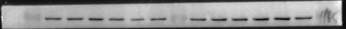 | NF-κB p65 | 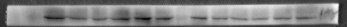 |
| Cl-Caspase-1 | 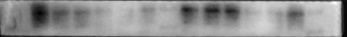 | Gsdmd | 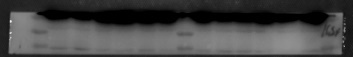 |
| β-actin | 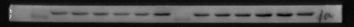 | β-actin | 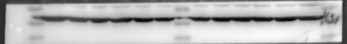 |
| Uncropped | 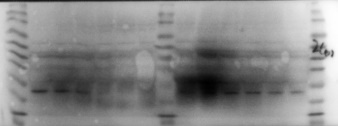  Cl-Caspase-1 | Uncropped | 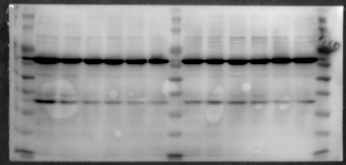  Gsdmd  Gsdmd-N |

Figure 5C

Uncropped

Gsdmd

Gsdmd-N


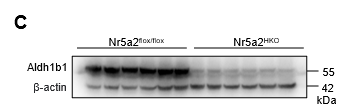


| 5C | Aldh1b1  β-actin | 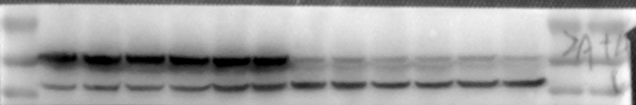 |
| --- | --- | --- |

Figure 6H and 6K


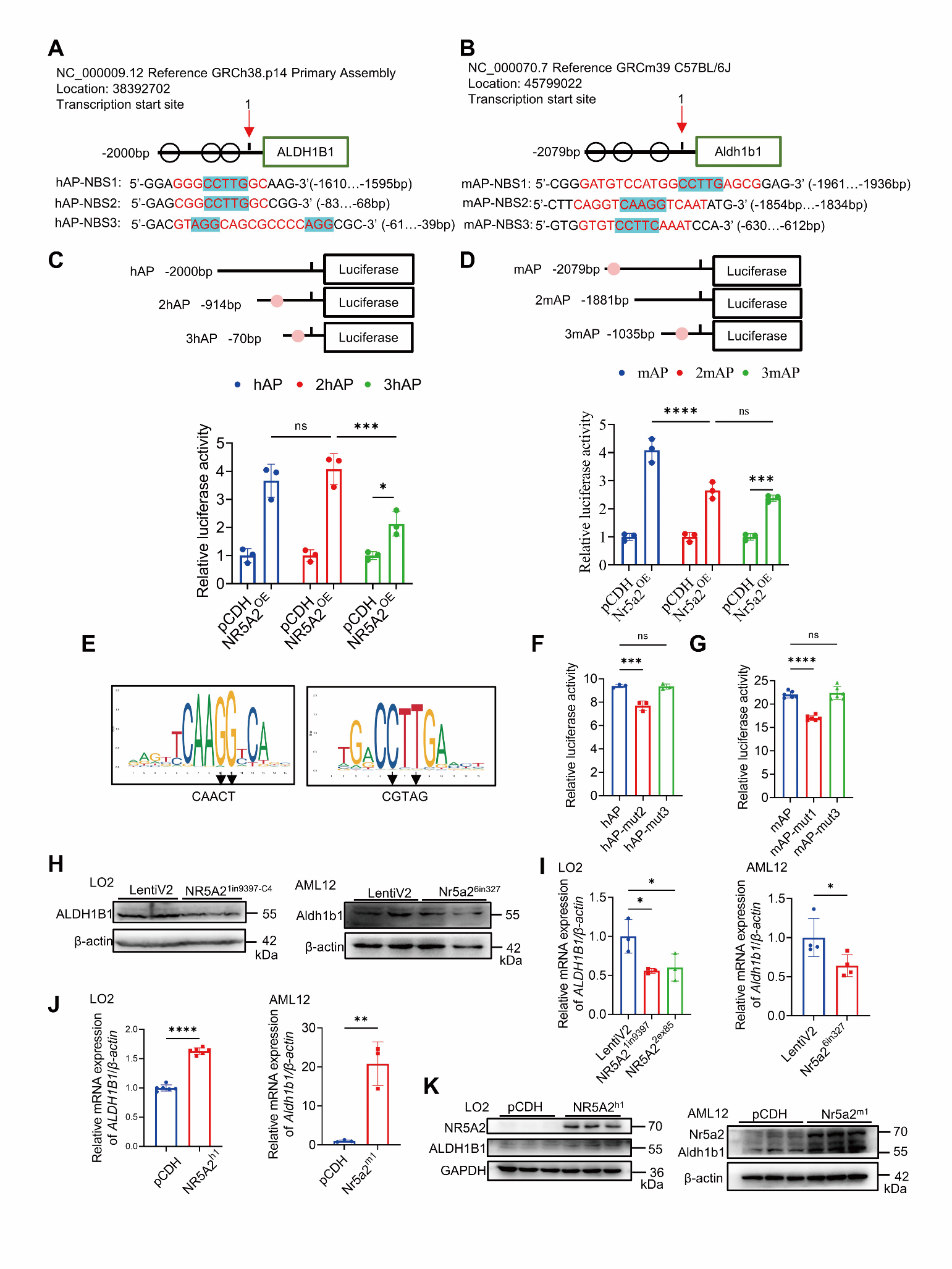

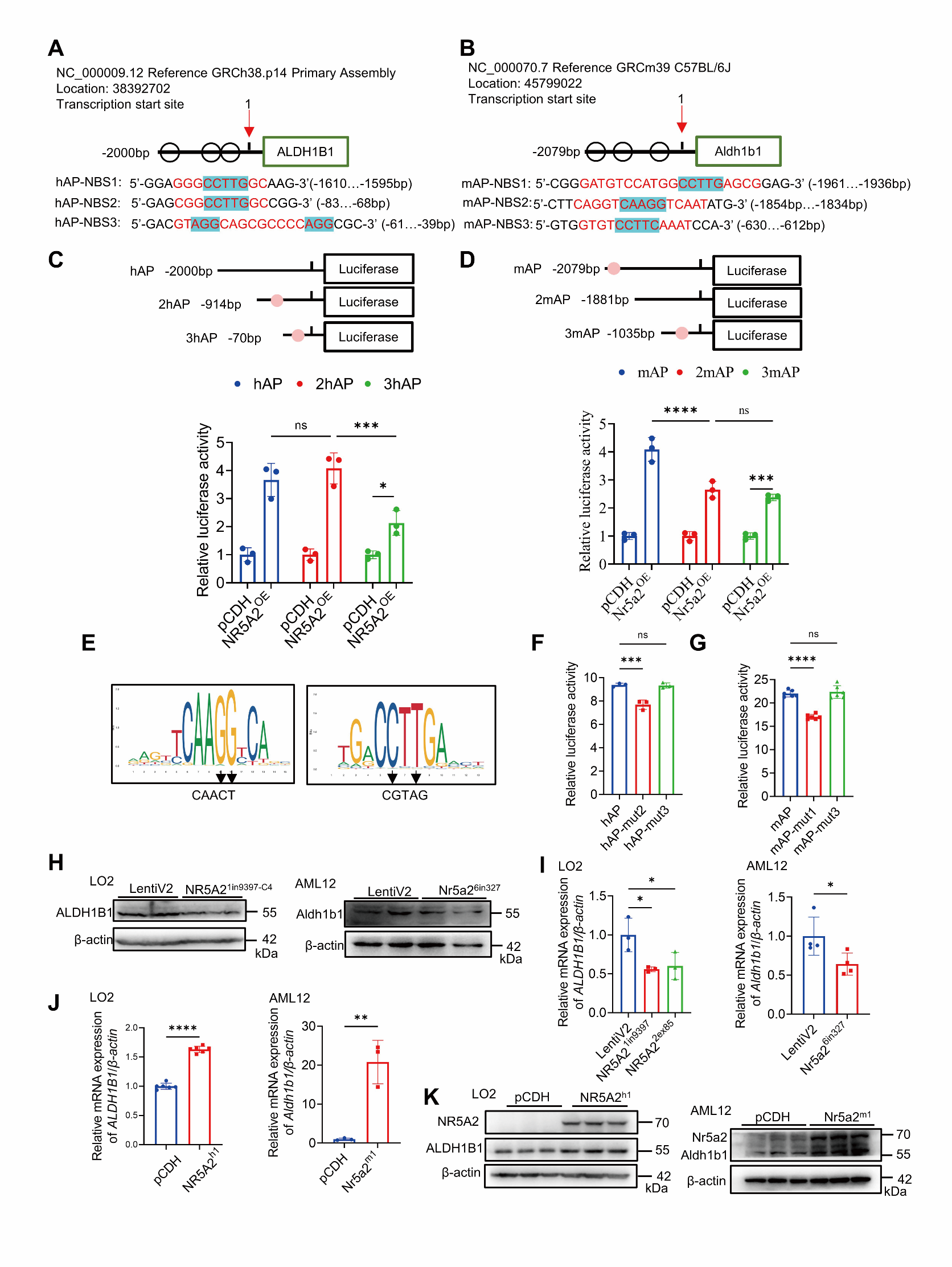


|  | 6H | | 6K | |
| --- | --- | --- | --- | --- |
| LO2 | ALDH1B1 | 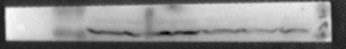 | ALDH1B1 | 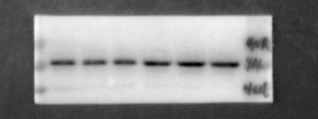 |
|  | β-actin | 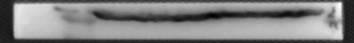 | NR5A2  β-actin | 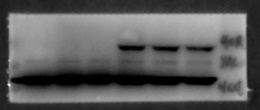 |
| AML12 | Aldh1b1 | 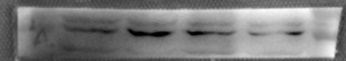 | Nr5a2  Aldh1b1 | 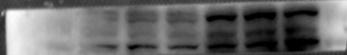 |
|  | β-actin | 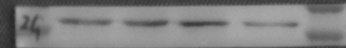 | β-actin | 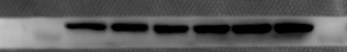 |

Figure 7A


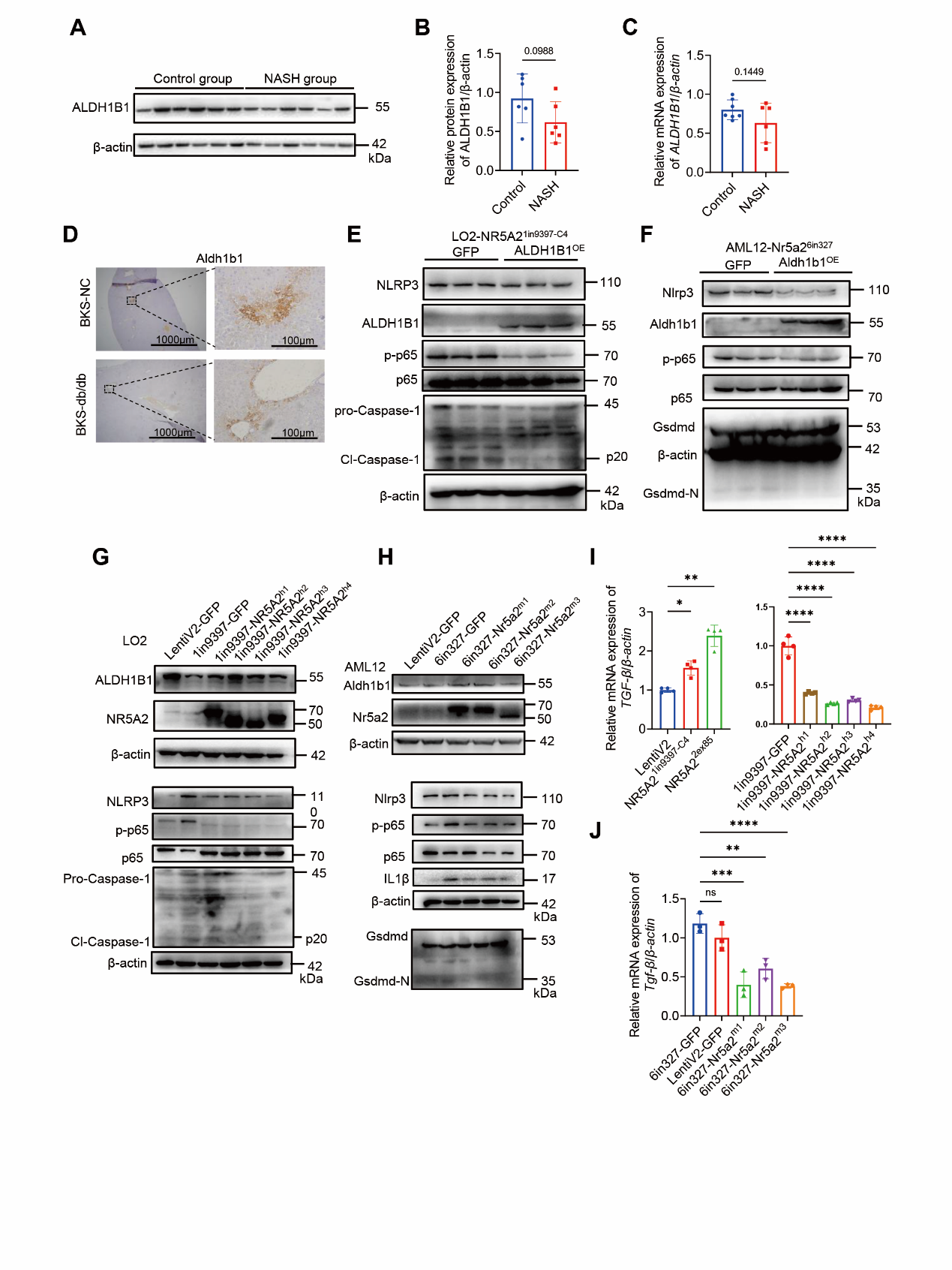


| 7A | Aldh1b1 | 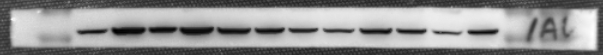 |
| --- | --- | --- |
|  | β-actin | 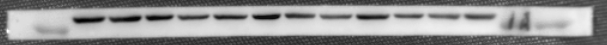 |

Figure 7E and 7F


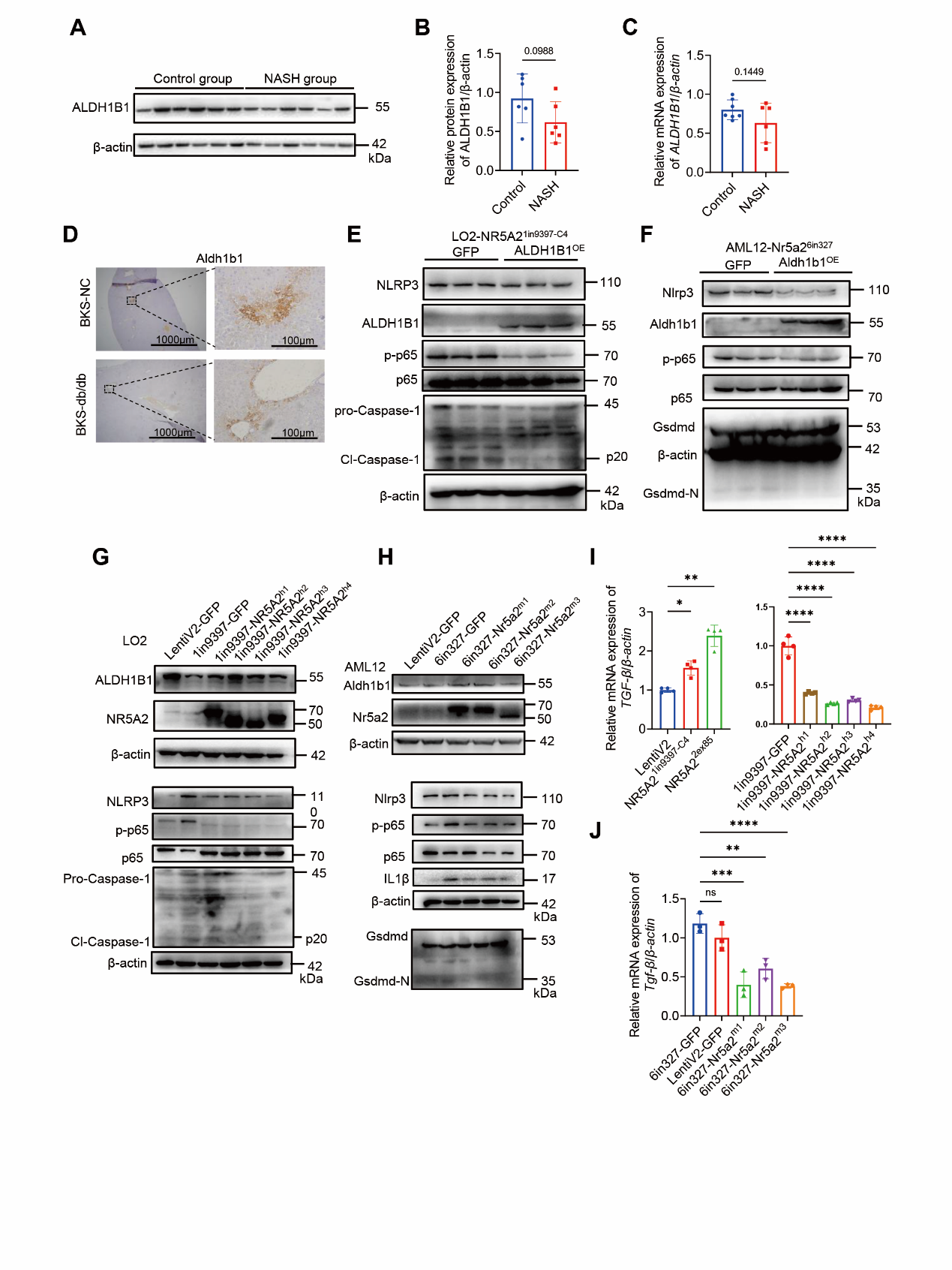


| 7E | | 7F | |
| --- | --- | --- | --- |
| NLRP3 | 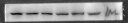 | Nlrp3 | 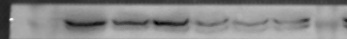 |
| ALDH1B1 | 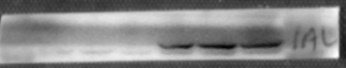 | Aldh1b1 | 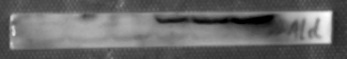 |
| NF-κB p-p65 | 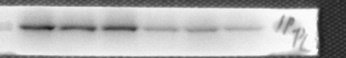 | NF-κB p-p65 | 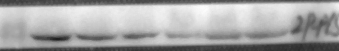 |
| NF-κB p65 | 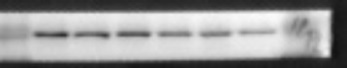 | NF-κB p65 | 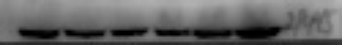 |
| Caspase-1 | 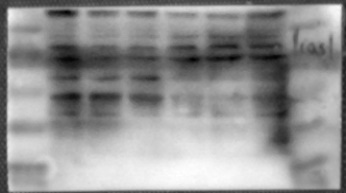 | Gsdmd  β-actin | 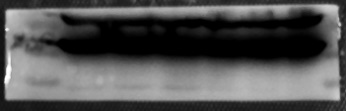 |
| β-actin | 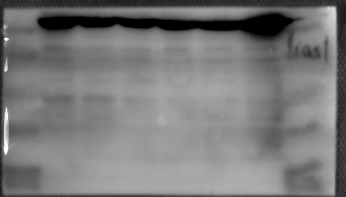 |  |  |
| Uncropped  ALDH1B1 | 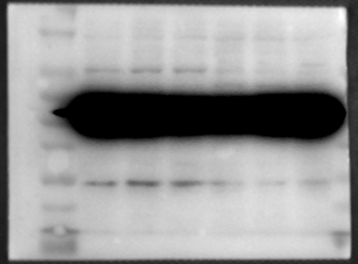 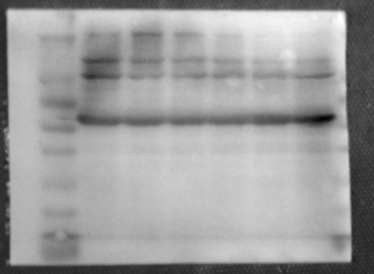  β-actin  Cl-Caspase-1  NLRP3  p65  p-p65 | | |

Figure 7G and 7H

β-actin


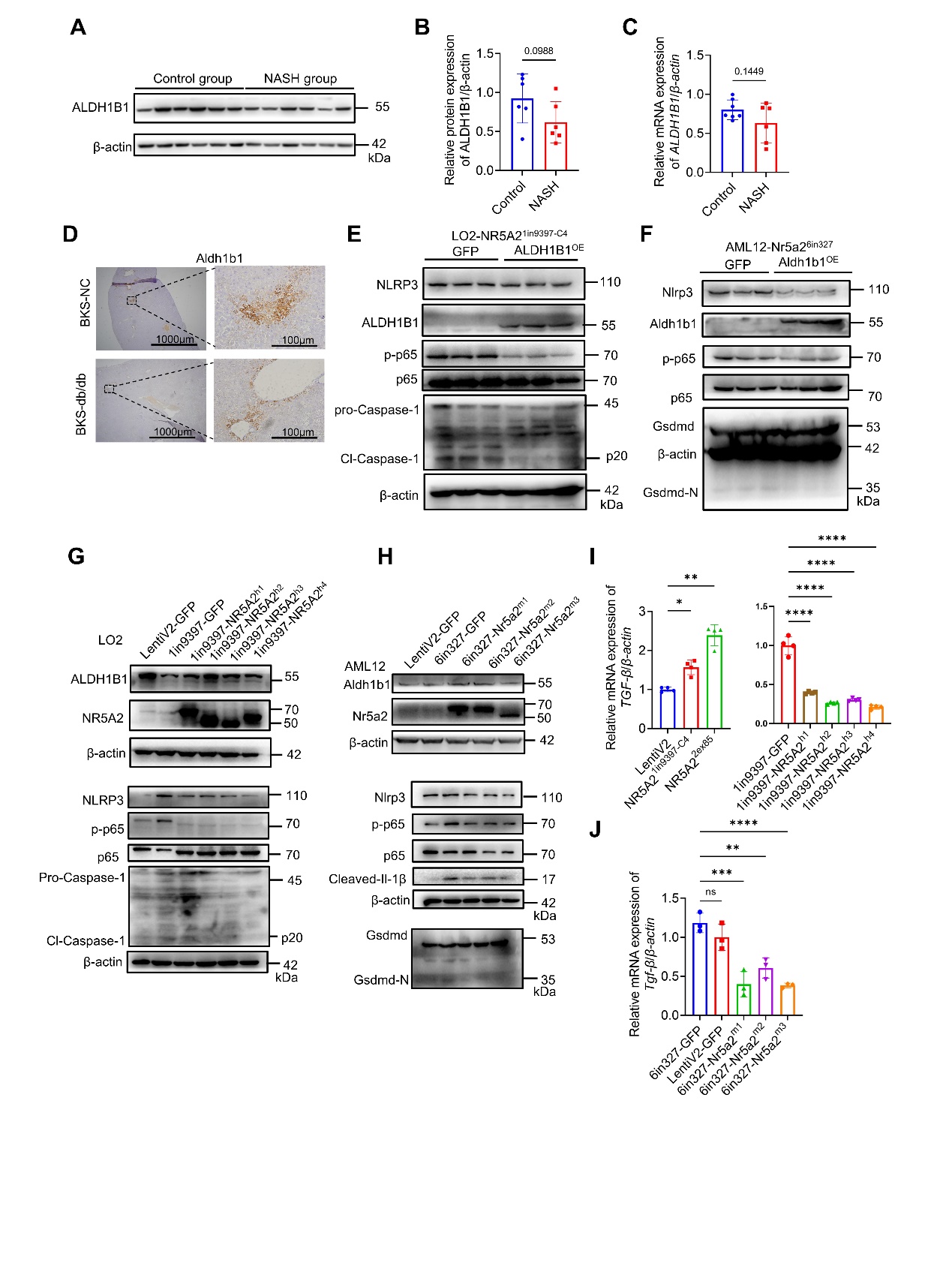


| 7G | | 7H | |
| --- | --- | --- | --- |
| ALDH1B1 | 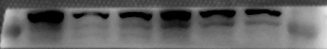 | Aldh1b1 | 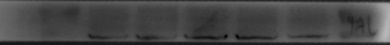 |
| NR5A2 | 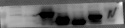 | Nr5a2 | 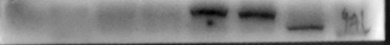 |
| β-actin | 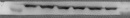 | β-actin | 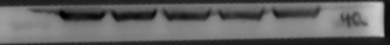 |
| NLRP3 | 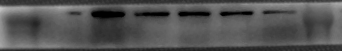 | Nlrp3 | **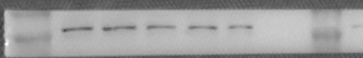** |
| NF-κB p-p65 | 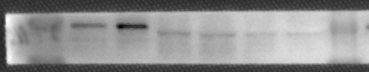 | NF-κB p-p65 | **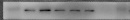** |
| NF-κB p65 | 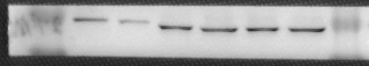 | NF-κB p65 | **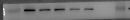** |
|  |  | Cleaved-Il-1β | **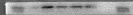** |
| β-actin | 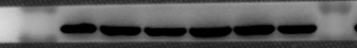 | β-actin | **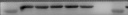** |
| Caspase-1 | 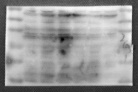 | Gsdmd | **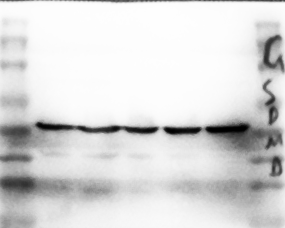** |
| Uncropped | 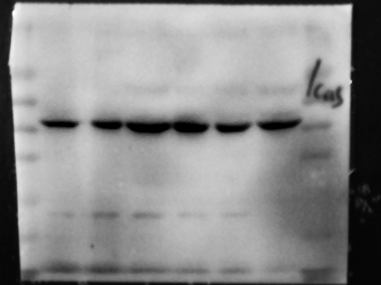  Cl-Caspase-1  β-actin  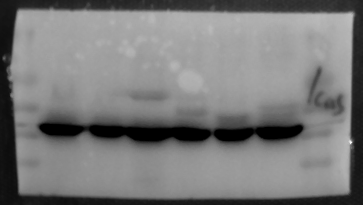  NR5A2  β-actin | Uncropped | **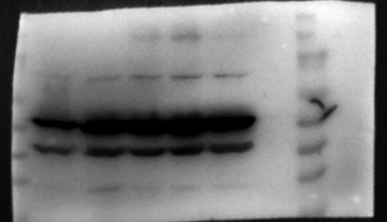**  β-actin  Gsdmd  Gsdmd-N  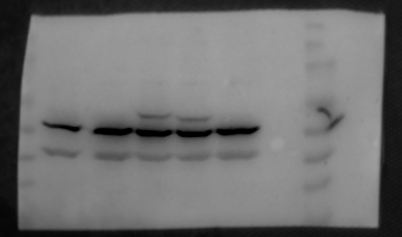  Gsdmd  Nr5a2  β-actin |

**Original western blots of supplemental figures**

Figure S1D and S1E


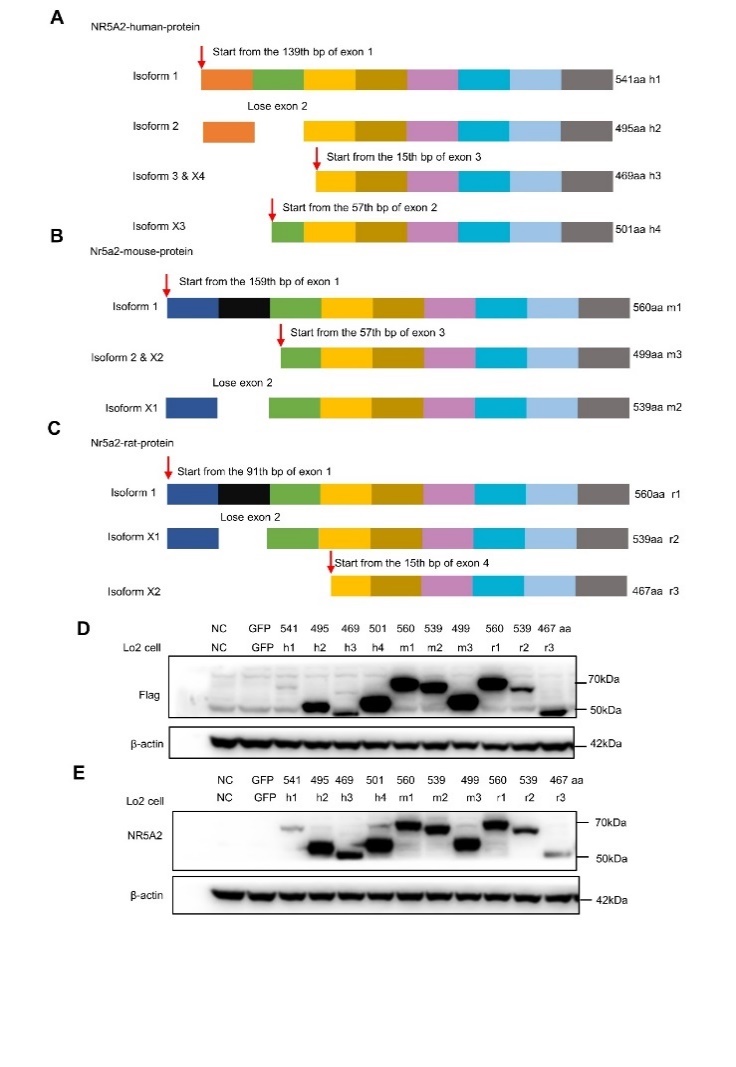

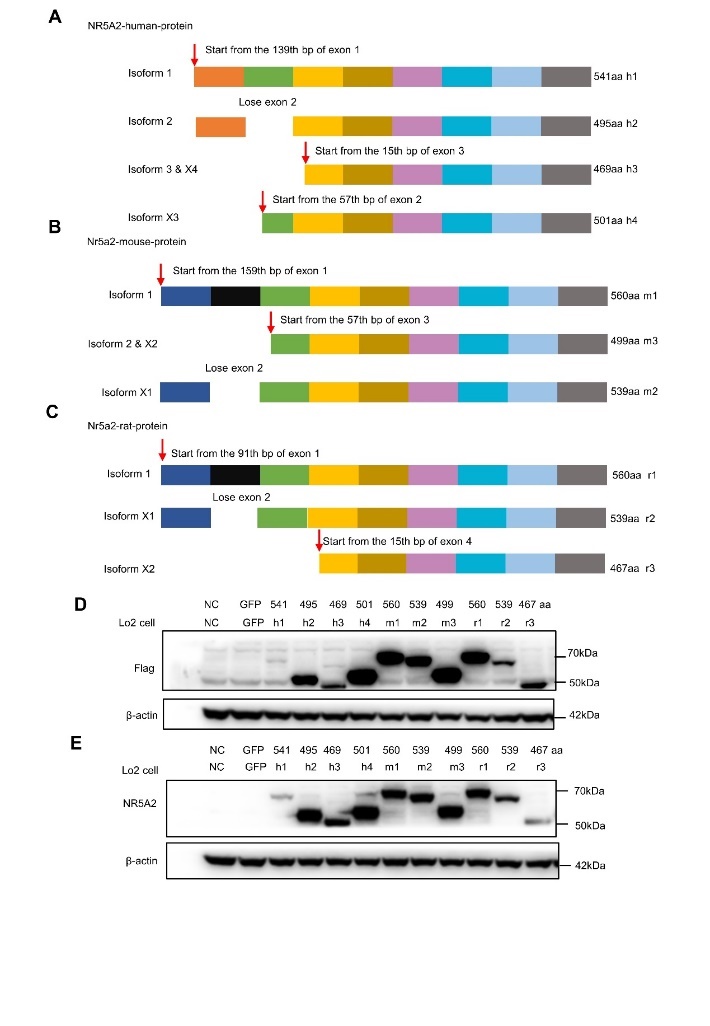


| S1D | Flag | 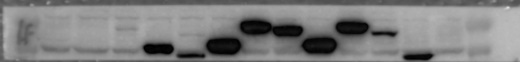 |
| --- | --- | --- |
|  | β-actin | 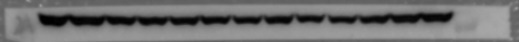 |
| S1E | NR5A2 | 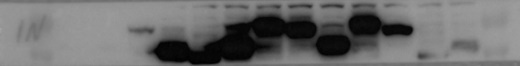 |
|  | β-actin | 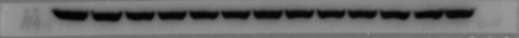 |

Figure S2G and S2I

| S2G | Nr5a2 |  |
| --- | --- | --- |
|  | Gapdh |  |
| S2I | Nr5a2 |  |
|  | β-actin |  |

Figure S4B, S4C and S4F

| S4B | | S4C | | S4F | |
| --- | --- | --- | --- | --- | --- |
| NR5A2 |  | NR5A2 |  | Nr5a2 |  |
| β-actin |  | β-actin |  | β-actin |  |

Figure S5A and S5B

| S5A | | S5B | |
| --- | --- | --- | --- |
| p-IKKα/β |  | TLR2 |  |
| IKKα |  | MYD88 |  |
| IKKβ |  | Cleaved-Caspase-1 |  |
| p-IκBα |  | β-actin |  |
| IκBα |  |  |  |
| Caspase-1 |  |  |  |
| β-actin |  |  |  |

Figure S6B and S6D

| S6B | | S6D | |
| --- | --- | --- | --- |
| ALDH1B1 |  | ALDH1B1 |  |
| NLRP3 |  | NLRP3 |  |
| NF-κB p-p65 |  | NF-κB p-p65 |  |
| NF-κB p65 |  | NF-κB p65 |  |
| Gapdh |  | p-IκBα |  |
|  |  | IκBα |  |
|  |  | p-IKKα/β |  |
|  |  | IKKβ |  |
|  |  | IKKα |  |
|  |  | β-actin |  |

Figure S6F

| S6F | ALDH1B1 |  |
| --- | --- | --- |
|  | β-actin |  |
